# Pancreatic alpha cell glucagon–liver FGF21 axis regulates beta cell regeneration in a mouse model of type 2 diabetes

**DOI:** 10.1007/s00125-022-05822-2

**Published:** 2022-11-04

**Authors:** Xiaona Cui, Jin Feng, Tianjiao Wei, Linxi Zhang, Shan Lang, Kun Yang, Jin Yang, Junling Liu, Michael Sterr, Heiko Lickert, Rui Wei, Tianpei Hong

**Affiliations:** 1 Department of Endocrinology and Metabolism, Peking University Third Hospital, Beijing, China; 2 Clinical Stem Research Cell Center, Peking University Third Hospital, Beijing, China; 3 Institute of Diabetes and Regeneration Research, Helmholtz Center Munich, Neuherberg, Germany; 4 German Center for Diabetes Research (DZD), Neuherberg, Germany; 5 School of Medicine, Technical University of Munich, Munich, Germany

**Keywords:** Alpha cell, Beta cell regeneration, FGF21, Glucagon receptor, Liver

## Abstract

**Aims/hypothesis:**

Glucagon receptor (GCGR) antagonism ameliorates hyperglycaemia and promotes beta cell regeneration in mouse models of type 2 diabetes. However, the underlying mechanisms remain unclear. The present study aimed to investigate the mechanism of beta cell regeneration induced by GCGR antagonism in mice.

**Methods:**

The *db/db* mice and high-fat diet (HFD)+streptozotocin (STZ)-induced mice with type 2 diabetes were treated with antagonistic GCGR monoclonal antibody (mAb), and the metabolic variables and islet cell quantification were evaluated. Plasma cytokine array and liver RNA sequencing data were used to screen possible mediators, including fibroblast growth factor 21 (FGF21). ELISA, quantitative RT-PCR and western blot were applied to verify FGF21 change. Blockage of FGF21 signalling by FGF21-neutralising antibody (nAb) was used to clarify whether FGF21 was involved in the effects of GCGR mAb on the expression of beta cell identity-related genes under plasma-conditional culture and hepatocyte co-culture conditions. FGF21 nAb-treated *db/db* mice, systemic *Fgf21*-knockout (*Fgf21*^−/−^) diabetic mice and hepatocyte-specific *Fgf21*-knockout (*Fgf21*^Hep−/−^) diabetic mice were used to reveal the involvement of FGF21 in beta cell regeneration. A BrdU tracing study was used to analyse beta cell proliferation in diabetic mice treated with GCGR mAb.

**Results:**

GCGR mAb treatment improved blood glucose control, and increased islet number (*db/db* 1.6±0.1 vs 0.8±0.1 per mm^2^, *p*<0.001; HFD+STZ 1.2±0.1 vs 0.5±0.1 per mm^2^, *p*<0.01) and area (*db/db* 2.5±0.2 vs 1.2±0.2%, *p*<0.001; HFD+STZ 1.0±0.1 vs 0.3±0.1%, *p*<0.01) in diabetic mice. The plasma cytokine array and liver RNA sequencing data showed that FGF21 levels in plasma and liver were upregulated by GCGR antagonism. The GCGR mAb induced upregulation of plasma FGF21 levels (*db/db* 661.5±40.0 vs 466.2±55.7 pg/ml, *p*<0.05; HFD+STZ 877.0±106.8 vs 445.5±54.0 pg/ml, *p*<0.05) and the liver levels of *Fgf21* mRNA (*db/db* 3.2±0.5 vs 1.8±0.1, *p*<0.05; HFD+STZ 2.0±0.3 vs 1.0±0.2, *p*<0.05) and protein (*db/db* 2.0±0.2 vs 1.4±0.1, *p*<0.05; HFD+STZ 1.6±0.1 vs 1.0±0.1, *p*<0.01). Exposure to plasma or hepatocytes from the GCGR mAb-treated mice upregulated the mRNA levels of characteristic genes associated with beta cell identity in cultured mouse islets and a beta cell line, and blockage of FGF21 activity by an FGF21 nAb diminished this upregulation. Notably, the effects of increased beta cell number induced by GCGR mAb were attenuated in FGF21 nAb-treated *db/db* mice, *Fgf21*^−/−^ diabetic mice and *Fgf21*^Hep−/−^ diabetic mice. Moreover, GCGR mAb treatment enhanced beta cell proliferation in the two groups of diabetic mice, and this effect was weakened in *Fgf21*^−/−^ and *Fgf21*^Hep−/−^ mice.

**Conclusions/interpretation:**

Our findings demonstrate that liver-derived FGF21 is involved in the GCGR antagonism-induced beta cell regeneration in a mouse model of type 2 diabetes.

**Graphical abstract:**

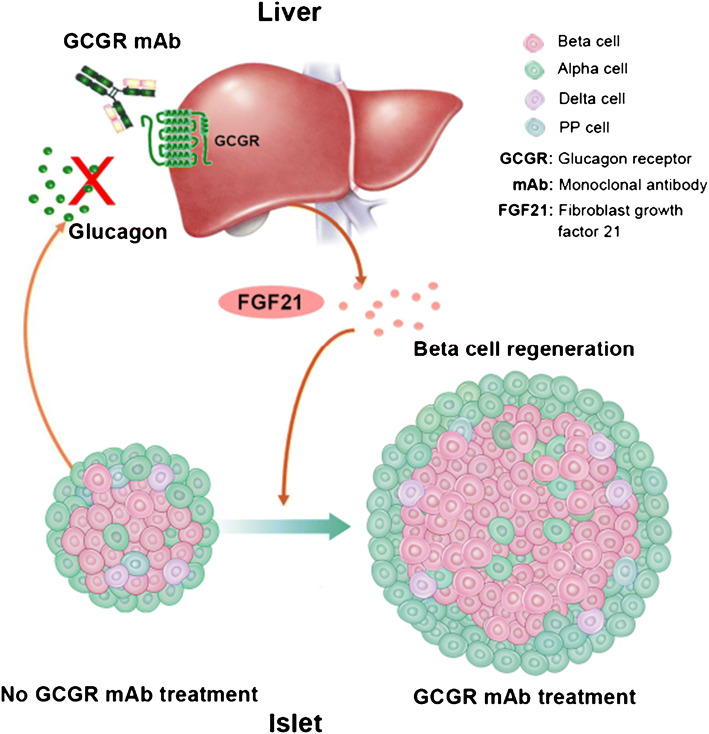

**Supplementary Information:**

The online version contains supplementary material available at 10.1007/s00125-022-05822-2.

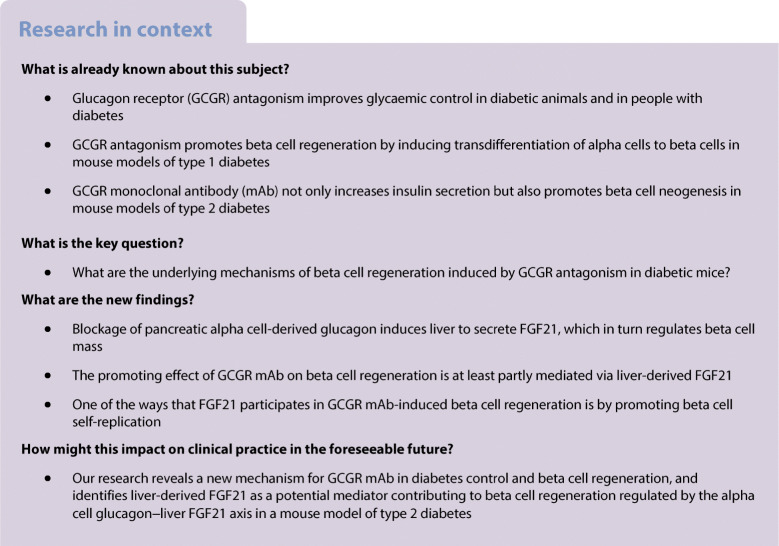



## Introduction

The prevalence of diabetes is increasing worldwide [1, 2]. Diabetes is a common non-communicable chronic disease arising from a dual hormone (insulin and glucagon) disorder [3–5]. Glucagon, a hormone secreted from pancreatic alpha cells, plays its physiological role by binding to glucagon receptor (GCGR) [6]. The principal target organ of glucagon is the liver, in which it promotes glycogen decomposition and gluconeogenesis thus elevating blood glucose levels [7]. Therefore, blockage of glucagon-GCGR signalling can be used as a glucose-lowering strategy. Antagonistic GCGR monoclonal antibody (mAb) has a strong hypoglycaemic effect in mouse models of type 1 diabetes and type 2 diabetes and in people with diabetes [8–11]. Surprisingly, we and other groups found that GCGR mAb promotes beta cell regeneration in diabetic mice [8, 10, 12]. However, the mechanisms underlying this regeneration remain unclear.

Several reports have demonstrated that the liver–islet axis participates in GCGR antagonism-induced alpha cell hyperplasia [13–17]. We supposed that the liver might also be involved in beta cell regeneration. The aims of this study were to identify the hepatokine induced by GCGR antagonism, and to clarify its role in beta cell regeneration. First, we investigated the effects of GCGR mAb on beta cell mass and function in two mouse models of type 2 diabetes. Next, we screened hepatokines by using the plasma cytokine array and liver mRNA sequencing data, and identified fibroblast growth factor 21 (FGF21) as a potential mediator. Then, we used FGF21-neutralising antibody (nAb) to clarify whether FGF21 was involved in the effects of GCGR mAb on the expression of beta cell identity-related markers under plasma-conditional culture and hepatocyte co-culture conditions. Furthermore, we evaluated the effects of GCGR mAb on beta cell regeneration in systemic *Fgf21*-knockout (*Fgf21*^−/−^) and hepatocyte-specific *Fgf21*-knockout (*Fgf21*^Hep−/−^) diabetic mice and in FGF21 nAb-treated *db/db* mice.

## Methods

For detailed methods, please refer to electronic supplementary material (ESM) Methods.

### Animal experiments

All animal experimental procedures were conducted at Peking University Health Science Center and were approved by the Institutional Animal Care and Use Committee. Eight-week-old male *db/db* mice (BKS-*Lepr*^em2Cd479^/Gpt [Strain no. T002407; GemPharmatech, Nanjing, China; https://www.gempharmatech.com/shop/detail/3913.html]) were used as a model of type 2 diabetes. Diabetes was induced in *Fgf21*^−/−^ (C57BL/6N-*Fgf21*^*em1Cya*^ [Cyagen Bioscience Company, Suzhou, China; https://www.cyagen.com/cn/zh-cn/sperm-bank-cn/S-KO-10895]) and *Fgf21*^Hep−/−^ mice (Alb-cre mice: B6.Cg-*Speer6-ps1*^*Tg(Alb-cre)21*^*Mgn*/J [the Jackson Laboratory, Barr Harbor, ME, USA; https://www.jax.org/strain/003574]; *Fgf21* Flox mice: B6.129S6(SJL)-*Fgf21*^*tm1.2Djm*^/J [the Jackson Laboratory; https://www.jax.org/strain/022361]) [18] and male C57BL/6N mice (Vital River Animal Center, Beijing, China) by high-fat diet (HFD) + streptozotocin (STZ).

Mice were treated for 6 weeks via weekly i.p. injection of 5 mg/kg REMD 2.59 (a human antagonistic GCGR mAb; REMD Biotherapeutics, Camarillo, CA, USA) or human IgG (as control). Mice were treated with 1 mg/ml BrdU in their drinking water for 7 days before being killed. To antagonise FGF21 activity, *db/db* mice were given i.p. injections of FGF21 nAb (Antibody & Immunoassay Services, Hong Kong, China) or rabbit IgG (as control) daily for 3 weeks at a dose of 6 μg/day.

### Glucose monitoring and hormone measurement

Blood glucose was measured by the glucose oxidase method using a OneTouch Ultra glucometer (LifeScan, Milpitas, CA, USA). To perform IPGTT, basal blood glucose levels were first measured after overnight fasting. Glucose solution (40% wt/vol.) was given by i.p. injection at a dose of 1 or 2 g/kg, and blood glucose levels were monitored at 30, 60 and 120 min after the glucose loading. If the blood glucose level was higher than 33.3 mmol/l (the upper detection limit of the glucometer), 33.3 mmol/l was recorded.

Specific ELISA kits were used to detect insulin (Millipore, Saint Charles, MO, USA), glucagon (R&D System, Minneapolis, MN, USA) and FGF21 (R&D system) following the manufacturers’ instructions.

### Proteome profiler array for mouse cytokines

Plasma samples from STZ-induced diabetic mice treated with GCGR mAb or human IgG (*n*=2) were analysed with a Mouse XL Cytokine Array Kit (R&D Systems) following the manufacturer’s instructions.

### Immunofluorescent staining and quantification

Pancreases were fixed with 10% (vol./vol.) neutral-buffered formalin and embedded in paraffin, and sections (5 μm thick) were prepared. Before testing, positive and negative controls were used to verify the antibodies. For immunofluorescence, sections were incubated with primary antibodies at 4°C overnight and secondary antibodies for 1 h at room temperature, followed by staining with DAPI. All primary and secondary antibodies were diluted in antibody dilution buffer (Tris-HCl buffer, BSA, sodium azide; Zhongshan Biotechnology, Beijing, China). Images were captured under Leica TCS SP8 confocal fluorescence microscope (Leica Microsystems, Wetzlar, Germany) or an automatic digital slide scanner (Pannoramic MIDI; 3DHISTECH, Budapest, Hungary).

For cell quantification in the immunofluorescent staining, three, four or five equally spaced sections (which covered the entire pancreas) per pancreas were imaged, the spacing between two adjacent sections was 200 μm and the total number of positive staining cells from three to six mice per group were counted manually.

### Beta cell line and primary islet experiments

Eight-week-old male C57BL/6N mice were treated with 5 mg/kg GCGR mAb or human IgG for 6 weeks. Mouse plasma was collected, and primary mouse hepatocytes were isolated. Mouse beta cell line (Min6 cells) or primary mouse islets were cultured with 10% mouse plasma and 90% medium, or co-cultured with hepatocytes for 24 h, with or without FGF21 nAb (10 μg/ml). Min6 cells were verified to be of mouse origin and negative for inter-species contamination from rat or human. Mycoplasma was tested as negative using Mycoplasma PCR detection kit (Beyotime Biotechnology, Shanghai, China).

### Quantitative RT-PCR and western blot

The RNA and proteins were collected, and quantitative RT-PCR and western blot were used to analyse gene and protein expression as previously described [9, 19]. The primer sequences are summarised in ESM Table 1. Before testing, positive and negative controls were used to verify the antibodies.

### Inclusion criteria and randomisation

Diabetic condition was defined if the fasting blood glucose level was ≥11.1 mmol/l for two consecutive measurements. Mice without diabetic condition were omitted from the study. Mice were randomised into groups having similar distributions based on their body weight and blood glucose level.

### Statistical analysis

Data are expressed as mean±SEM or median (IQR). All statistical analyses were performed using GraphPad Prism v.7.0 (GraphPad Software, San Diego, CA, USA). Statistical significance was defined as *p*<0.05 and determined by ANOVA followed by the post hoc Tukey–Kramer test, Student’s *t* test or Mann–Whitney test, as appropriate. Blinding was not carried out.

## Results

### GCGR mAb ameliorates hyperglycaemia and promotes islet regeneration in diabetic mice

**Fig. 1:**
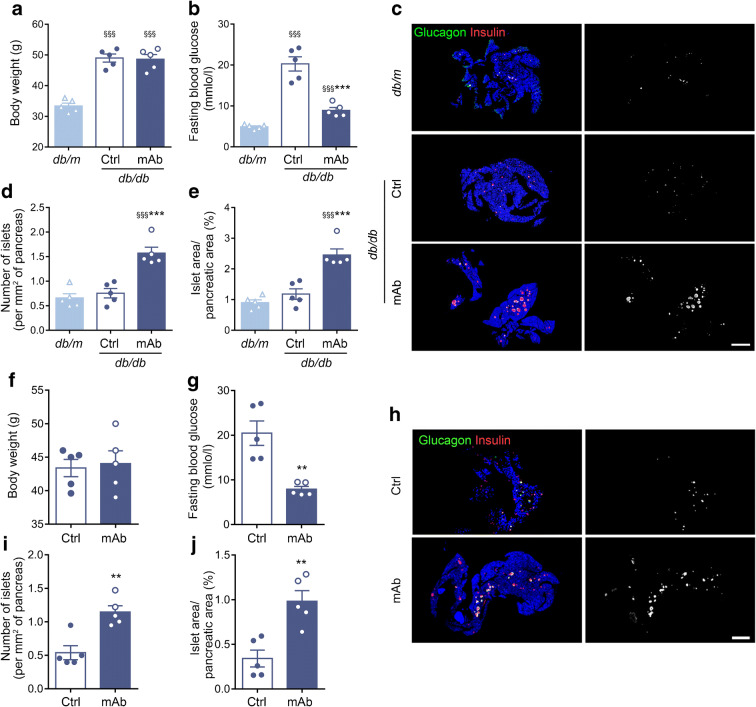
Metabolic variables and pancreatic histological analysis in two mouse models of type 2 diabetes following treatment with GCGR mAb or human IgG control for 6 weeks. (**a**–**e**) Variables in *db/db* mice. Age-matched *db/m* mice treated with human IgG were included as a normal control. (**f**–**j**) Variables in HFD+STZ-induced diabetic mice. (**a**, **f**) Body weight. (**b**, **g**) Fasting blood glucose. (**c**, **h**) Representative images of the whole pancreases of the *db/db* mice (**c**) and HFD+STZ-induced diabetic mice (**h**) immunostained for glucagon and insulin, together with monochrome images of immunostaining in the same tissues with the cells immunolabelled positively for either glucagon or insulin displayed in white. Scale bar, 2000 μm. (**d**, **e**) Quantification of islet number (**d**) and islet area (**e**) in *db/db* mice. (**i**, **j**) Quantification of islet number (**i**) and islet area (**j**) in HFD+STZ-induced diabetic mice. *n*=5 mice per group. Data represent the mean±SEM. **p<0.01, ***p<0.001 vs control group; ^§§§^*p*<0.001 vs *db/m* mice (ANOVA followed by post hoc Tukey–Kramer test, or Student’s *t* test, as appropriate). Ctrl, control group

Compared with IgG control, GCGR mAb had little effect on body weight (Fig. 1a,f) but significantly lowered fasting blood glucose level in *db/db* mice and HFD+STZ-induced diabetic mice (Fig. 1b,g). Notably, GCGR mAb induced a remarkable increase in islet number (*db/db* 1.6±0.1 vs 0.8±0.1 per mm^2^, *p*<0.001; HFD+STZ 1.2±0.1 vs 0.5±0.1 per mm^2^, *p*<0.01) and islet area (*db/db* 2.5±0.2 vs 1.2±0.2%, *p*<0.001; HFD+STZ 1.0±0.1 vs 0.3±0.1%, *p*<0.01) in the two mouse models of type 2 diabetes (Fig. 1c–e,h–j).

### Soluble factors from plasma and liver of the GCGR mAb-treated mice regulate beta cell identity in vitro

To explore the possible mechanisms underlying the GCGR mAb-mediated regulation of beta cells, we collected plasma from the GCGR mAb- or IgG-treated mice, and used the plasma for the conditioned culture of mouse beta cell line Min6 cells or primary mouse islets. Results showed that exposure to plasma of the GCGR mAb-treated mice upregulated the expression of *Ins1* (encodes insulin 1), *Ins2* (encodes insulin 2), *Pcsk1* (encodes prohormone convertase 1/3) and *Pdx1* (encodes pancreatic and duodenal homeobox 1 [PDX1]) in Min6 cells (ESM Fig. 1a), and increased the mRNA levels of *Gcg* (encodes proglucagon) and beta cell characteristic genes, including *Ins1*, *Ins2*, *Pcsk1* and *Pdx1*, in mouse islets (ESM Fig. 1b).

The liver–alpha cell axis plays a pivotal role in GCGR mAb-induced alpha cell proliferation [13]. We supposed that factors released from the liver might also participate in beta cell regulation. Therefore, we isolated primary hepatocytes from the GCGR mAb- or IgG-treated mice, and co-cultured these hepatocytes with Min6 cells or mouse islets. Results showed that co-culture with hepatocytes from the GCGR mAb-treated mice upregulated the expression of *Ins1*, *Ins2*, *Pcsk1* and *Pdx1* in Min6 cells (ESM Fig. 1c), and increased the mRNA levels of *Gcg*, *Ins1*, *Ins2*, *Pcsk1* and *Pdx1* in mouse islets (ESM Fig. 1d). Collectively, these results suggested that soluble factors derived from the plasma and hepatocytes of GCGR mAb-treated mice could regulate beta cell identity in vitro.

### Plasma and liver FGF21 levels are upregulated by GCGR mAb in mice

**Fig. 2:**
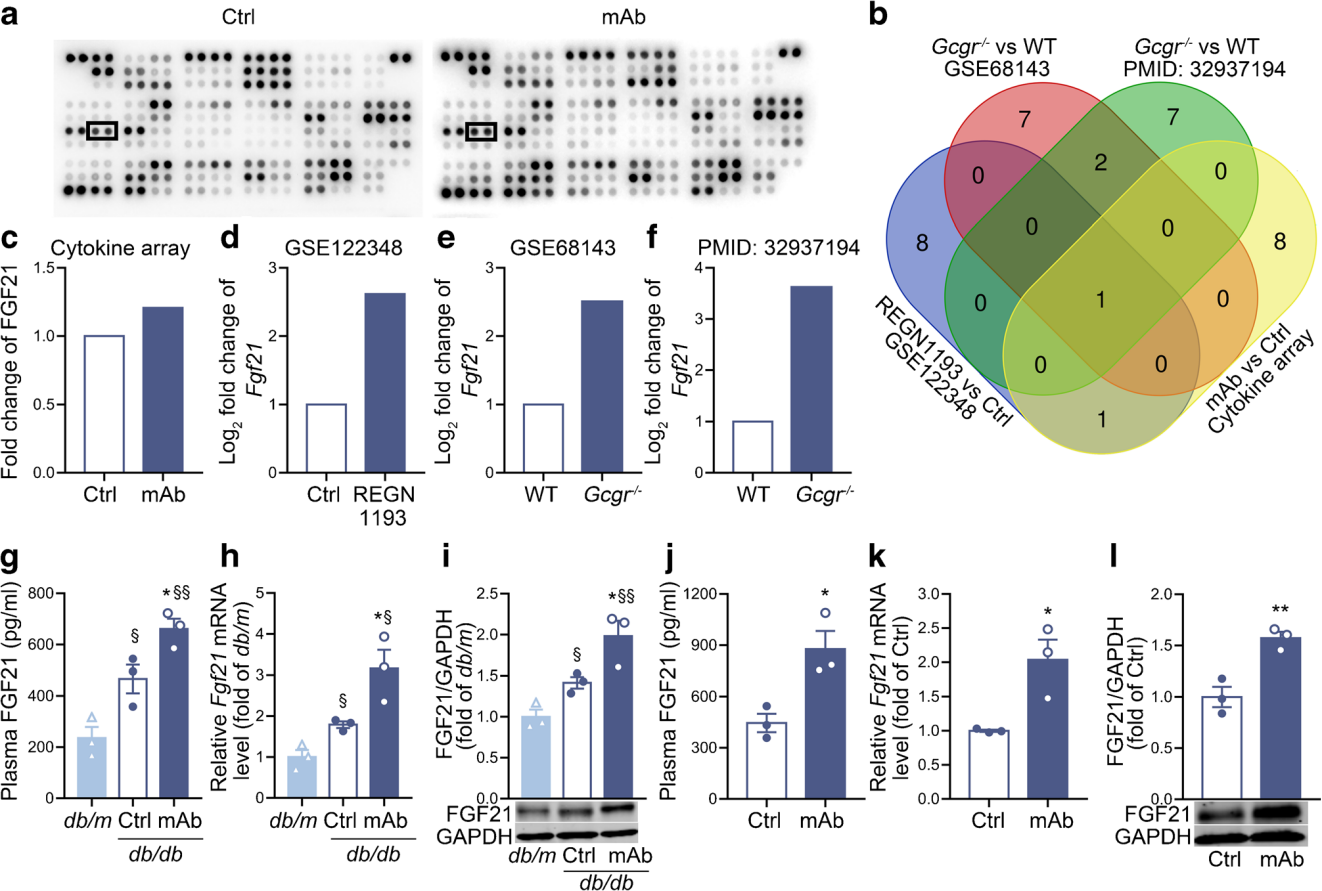
Screening and identification of liver-derived FGF21. (**a**) Cytokine array screening for the change in cytokine levels in the plasma of STZ-induced diabetic mice treated with GCGR mAb (*n*=2) or human IgG control (*n*=2). Cytokine profiles in two independent experiments. The boxes indicate blots of FGF21. (**b**) Venn diagram of the top ten upregulated proteins or genes based on the plasma cytokine array or liver mRNA sequencing data from four datasets. Dataset 1, cytokine array screening data in this study; dataset 2, liver mRNA sequencing data in the GCGR antagonist REGN1193-treated mice and control mice (NCBI database GSE122348); dataset 3, liver mRNA sequencing data in the *Gcgr*-knockout (*Gcgr*^−/−^) mice and WT mice (NCBI database GSE68143); dataset 4, liver mRNA sequencing data in the *Gcgr*^−/−^ mice and WT mice in the published article [20] (PMID 32937194). FGF21 was the only molecule common to the four datasets. (**c**–**f**) Relative change of FGF21 level in these four datasets. (**g**–**i**) Plasma FGF21 (**g**), liver FGF21 mRNA (**h**) and protein (**i**) levels in *db/db* mice treated with GCGR mAb or human IgG control for 6 weeks. (**j**–**l**) Plasma FGF21 (**j**), liver FGF21 mRNA (**k**) and protein (**l**) levels in HFD+STZ-induced diabetic mice treated with GCGR mAb or human IgG control for 6 weeks. *n*=3 mice per group. Data are expressed as mean±SEM. *p<0.05, **p<0.01 vs control group; ^§^*p*<0.05, ^§§^*p*<0.01 vs *db/m* mice (ANOVA followed by post hoc Tukey–Kramer test, or Student’s *t* test, as appropriate). Ctrl, control group

To identify the potential soluble factor that was derived from hepatocytes and secreted into plasma, we used a cytokine array kit to assess plasmatic cytokine profiles, and found ten cytokines that were upregulated >1.2-fold in the GCGR mAb-treated mice (Fig. 2a and ESM Table 2). We also analysed the liver RNA sequencing data in the public databases GSE68143 and GSE122348, and in the article published by Winther-Sorensen et al [20]. The liver RNA profiles in *Gcgr*-knockout or GCGR antagonist-treated mice showed a striking difference as compared with wild-type (WT) or control-treated mice, respectively. The ten proteins/genes with the highest fold increase in each dataset are shown in ESM Fig. 2. Notably, FGF21 was the only common factor observed both in our cytokine array data and in the gene expression profiles (Fig. 2b–f).

Subsequently, we confirmed the GCGR antagonism-induced upregulation of FGF21. ELISA analysis showed that, compared with IgG control, plasma FGF21 level was upregulated by GCGR mAb in *db/db* mice (661.5±40.0 vs 466.2±55.7 pg/ml, *p*<0.05) and HFD+STZ-induced diabetic mice (877.0±106.8 vs 445.5±54.0 pg/ml, *p*<0.05) (Fig. 2g,j). The levels of *Fgf21* mRNA (*db/db* 3.2±0.5 vs 1.8±0.1, *p*<0.05; HFD+STZ 2.0±0.3 vs 1.0±0.2, *p*<0.05) and protein (*db/db* 2.0±0.2 vs 1.4±0.1, *p*<0.05; HFD+STZ 1.6±0.1 vs 1.0±0.1, *p*<0.01) in liver tissues were augmented by GCGR mAb in the two groups of diabetic mice (Fig. 2h,i,k,l). Similarly, FGF21 levels in plasma and liver were also increased by GCGR mAb in non-diabetic C57BL6/N mice (ESM Fig. 3).

### Plasma and liver FGF21 participates in the GCGR mAb-mediated regulation of beta cell identity in vitro

Activation of FGF receptor (FGFR) by FGF21 is dependent on the transmembrane protein β-klotho (KLB), and the function of FGF21 is mediated mainly via FGFR1c/KLB and FGFR3c/KLB [21–23]. To ascertain whether plasma and liver FGF21 could act on endocrine pancreas, we analysed the single-cell RNA sequencing data of in vitro differentiation of beta cells [24] (available in the public database GSE114412). The analysis showed that in the later stages of in vitro islet cell differentiation, *FGFR1* was highly expressed in pancreatic endocrine cells, including alpha cells, beta cells and neurogenin 3-positive pancreatic endocrine progenitors (ESM Figs 4 and 5), suggesting that FGF21 might exert an effect on endocrine pancreas.

**Fig. 3:**
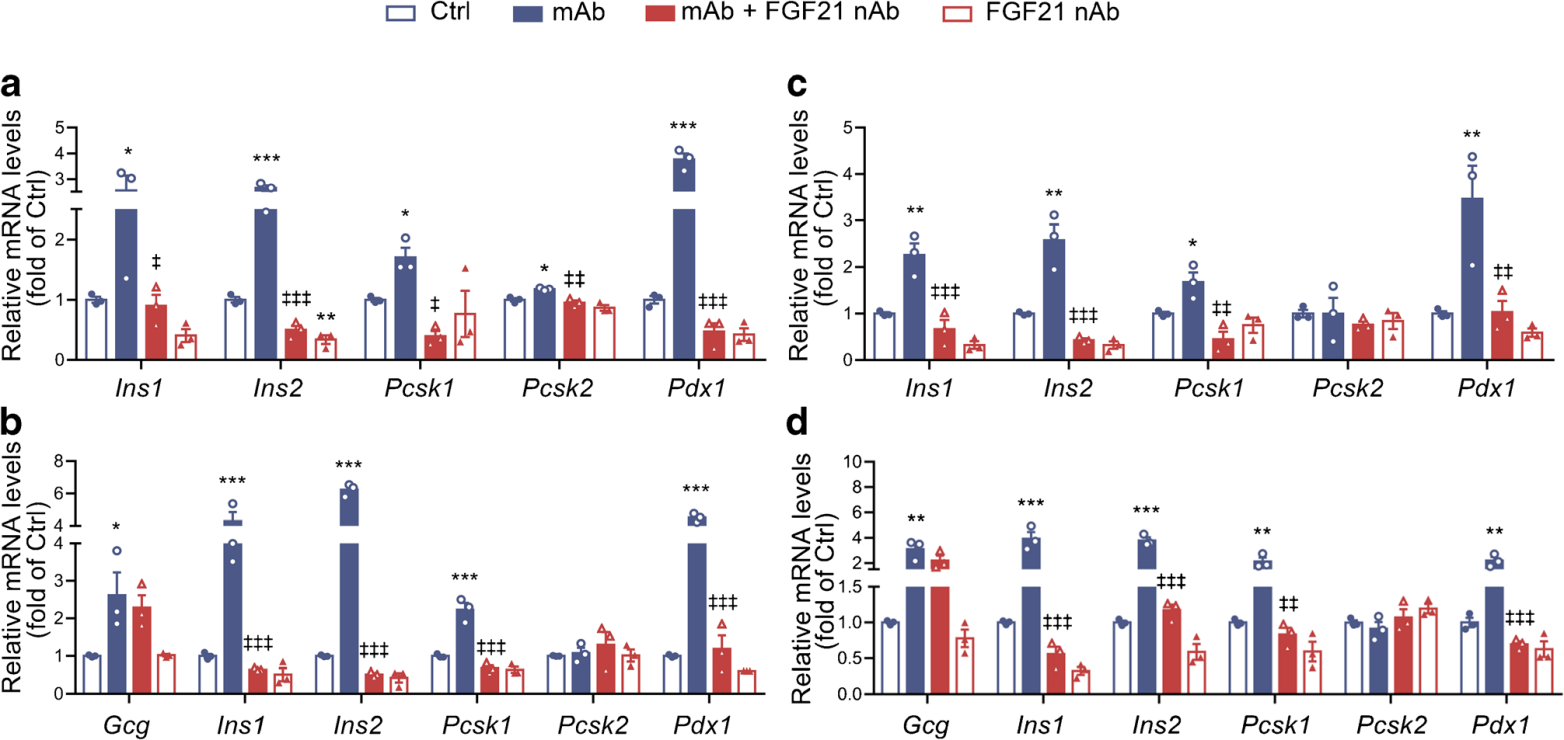
Regulation of beta cell identity by GCGR mAb in vitro. (**a**–**d**) Mouse beta cell line Min6 cells (**a**, **c**) and primary mouse islets (**b**, **d**) were exposed to plasma (**a**, **b**) or hepatocytes (**c**, **d**) from GCGR mAb-treated non-diabetic C57BL/6N mice, in the absence or presence of an FGF21 nAb for 24 h. Relative gene expression was detected by quantitative RT-PCR. Data represent three independent experiments and are expressed as mean±SEM. *p<0.05, **p<0.01, ***p<0.001 vs control group; ^‡^*p*<0.05, ^‡‡^*p*<0.01, ^‡‡‡^*p*<0.001 vs GCGR mAb group (ANOVA followed by post hoc Tukey–Kramer test). Ctrl, control group

Subsequently, we wanted to clarify whether the upregulated FGF21 level was involved in the GCGR mAb-induced regulation of beta cell identity. In the plasma-conditional culture experiments, the upregulated mRNA levels of *Ins1*, *Ins2*, *Pcsk1* and *Pdx1* in either Min6 cells or mouse islets induced by exposure to plasma of the GCGR mAb-treated mice were attenuated by addition of FGF21 nAb (Fig. 3a,b). In the hepatocyte co-culture studies of Min6 cells or mouse islets, FGF21 nAb displayed similar effects (Fig. 3c,d). These results proved that liver-derived FGF21 participated in the GCGR mAb-mediated regulation of beta cell identity in vitro.

### Systemic FGF21 participates in GCGR mAb-induced beta cell regeneration in vivo

**Fig. 4:**
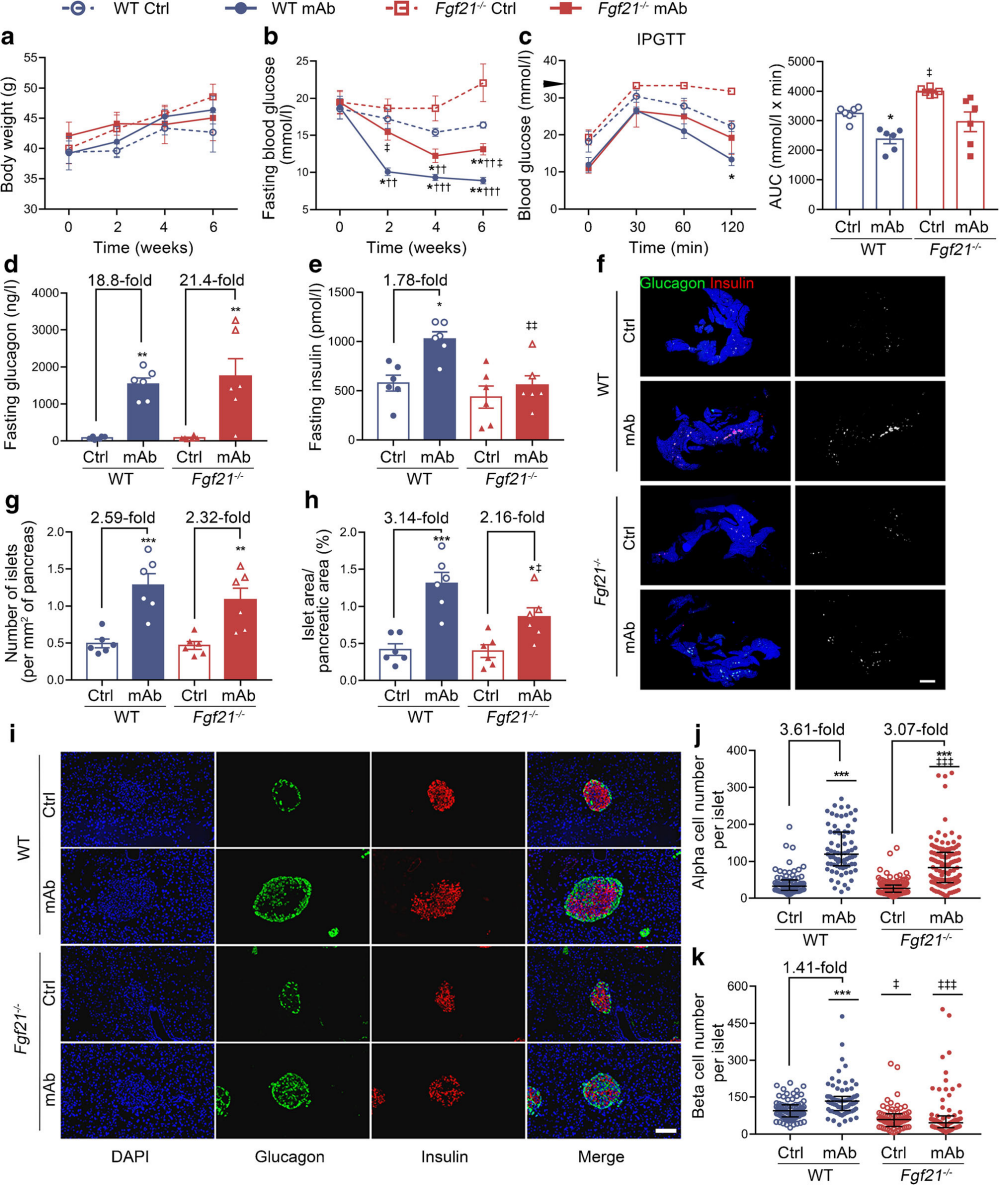
Metabolic variables, hormone levels and pancreatic histological analysis in systemic *Fgf21*-knockout diabetic mice treated with GCGR mAb or human IgG control for 6 weeks. (**a**) Body weight. (**b**) Fasting blood glucose. (**c**) Blood glucose during the IPGTT (black triangle indicates 33.3 mmol/l, the upper detection limit of the glucometer), and the AUC for blood glucose during the IPGTT. (**d**) Fasting plasma glucagon. (**e**) Fasting plasma insulin. (**f**) Histological analysis of the pancreas, showing representative images of the whole pancreas immunostained for glucagon and insulin together with monochrome images of immunostaining in the same tissues (cells immunolabelled positively for either glucagon or insulin are displayed in white). Scale bar, 2000 μm. (**g**, **h**) Quantification of islet number (**g**) and islet area (**h**). *n*=6 mice per group. (**i**) Representative images of islets immunostained for glucagon and insulin in the pancreatic tissues. (**j**, **k**) Quantification of alpha cell number (**j**) and beta cell number (**k**) per islet slice. Scale bar, 50 μm. Eighteen sections of *n*=6 mice per group. Data are expressed as the mean±SEM or median (IQR). *p<0.05, **p<0.01, ***p<0.001 vs IgG control in the same genotype; ^††^*p*<0.01, ^†††^*p*<0.001 vs pre-treatment in the same group; ^‡^*p*<0.05, ^‡‡^*p*<0.01, ^‡‡‡^*p*<0.001 vs WT group on the same treatment (ANOVA followed by post hoc Tukey–Kramer test, or Mann–Whitney test, as appropriate). Ctrl, control group

To determine whether FGF21 plays a role in GCGR mAb-induced beta cell regeneration in vivo, *Fgf21*^−/−^ and WT diabetic mice were treated with GCGR mAb or IgG. Body weight was comparable among the four groups (Fig. 4a). GCGR mAb significantly lowered fasting blood glucose level and improved glucose tolerance in WT and *Fgf21*^−/−^ mice (Fig. 4b,c), indicating that FGF21 was not a key mediator in the GCGR mAb-induced hypoglycaemic effect. *Fgf21*^−/−^ mice displayed higher fasting blood glucose and poorer glucose tolerance as compared with WT mice on the same treatment (Fig. 4b,c), suggesting that FGF21 itself participated in glucose homeostasis. Compared with IgG control treatment, fasting glucagon level was increased by GCGR mAb in WT mice (1537.0±165.7 vs 81.9±16.0 ng/l, *p*<0.01) and *Fgf21*^−/−^ mice (1744.0±480.8 vs 81.1±17.2 ng/l, *p*<0.01) (Fig. 4d). The fold change induced by GCGR mAb was comparable between *Fgf21*^−/−^ and WT mice, suggesting that FGF21 did not affect the GCGR mAb-mediated upregulation of glucagon secretion. GCGR mAb upregulated plasma insulin levels in WT mice (1026.0±72.3 vs 577.8±80.3 pmol/l, *p*<0.05) but this increment disappeared in *Fgf21*^−/−^ mice (554.7±97.0 vs 435.9±112.2 pmol/l, *p*=0.80) (Fig. 4e), indicating that FGF21 participated in the upregulating effect of insulin secretion induced by GCGR mAb. Compared with IgG control, GCGR mAb increased islet numbers in WT mice (1.3±0.2 vs 0.5±0.1 per mm^2^, *p*<0.001) and *Fgf21*^−/−^ mice (1.1±0.2 vs 0.5±0.1 per mm^2^, *p*<0.01), with no difference in the fold change between *Fgf21*^−/−^ and WT mice (Fig. 4f,g). GCGR mAb increased islet area (1.3±0.2 vs 0.4±0.1%, *p*<0.001), alpha cell number (119.0 [89.0–179.0] vs 33.0 [21.5–49.0], *p*<0.001) and beta cell number (133.0 [96.0–152.0] vs 94.0 [70.5–118.0], *p*<0.001) in WT mice (Fig. 4f,h–k). In *Fgf21*^−/−^ mice, GCGR mAb could still increase islet area (0.9±0.1 vs 0.4±0.1%, *p*<0.05) and alpha cell number (83.0 [43.0–124.0] vs 27.0 [16.0–36.0], *p*<0.001), albeit these increments were smaller in *Fgf21*^−/−^ mice than in WT mice. Notably, GCGR mAb could not increase beta cell number (46.0 [26.0–71.5] vs 58.0 [31.0–81.0], *p*=0.16) in *Fgf21*^−/−^ mice (Fig. 4i,k). These results suggest that FGF21 is involved in the promoting effects of GCGR mAb on islet regeneration, especially beta cell regeneration.

**Fig. 5:**
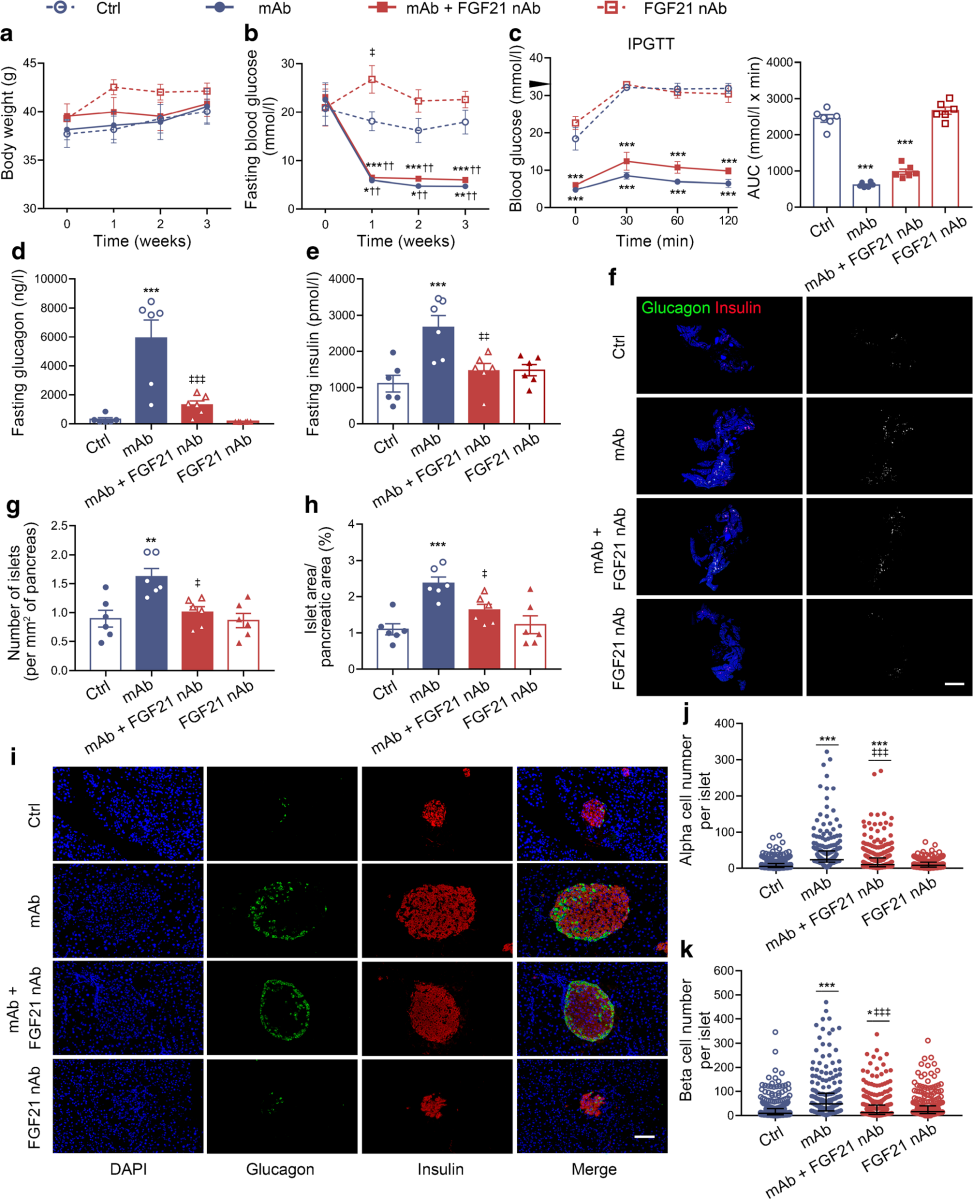
Metabolic variables, hormone levels and pancreatic histological analysis in *db/db* mice treated with GCGR mAb and/or FGF21 nAb for 3 weeks. (**a**) Body weight. (**b**) Fasting blood glucose. (**c**) Blood glucose during the IPGTT (black triangle indicates 33.3 mmol/l, the upper detection limit of the glucometer), and the AUC for blood glucose during the IPGTT. (**d**) Fasting plasma glucagon. (**e**) Fasting plasma insulin. (**f**) Histological analysis of the pancreas, showing representative images of the whole pancreas immunostained for glucagon and insulin together with monochrome images of immunostaining in the same tissues (cells immunolabelled positively for either glucagon or insulin are displayed in white). Scale bar, 2000 μm. (**g**, **h**) Quantification of islet number (**g**) and islet area (**h**). *n*=6 mice per group. (**i**) Representative images of islets immunostained for glucagon and insulin in the pancreatic tissues. (**j**, **k**) Quantification of alpha cell number (**j**) and beta cell number (**k**) per islet slice. Scale bar, 50 μm. Eighteen sections of *n*=6 mice per group. Data are expressed as the mean±SEM or median (IQR). *p<0.05, **p<0.01, ***p<0.001 vs control group; ^††^*p*<0.01 vs pre-treatment in the same group; ^‡^*p*<0.05, ^‡‡^*p*<0.01, ^‡‡‡^*p*<0.001 vs GCGR mAb group (ANOVA followed by post hoc Tukey–Kramer test, or Mann–Whitney test, as appropriate). Ctrl, control group

To exclude the effect of constitutive *Fgf21* knockout on islet development, we used FGF21 nAb for non-genetic blockage of FGF21 in *db/db* mice, and verified the involvement of FGF21 in GCGR mAb-induced beta cell regeneration. Body weight among the four groups (control, GCGR mAb, GCGR mAb+FGF21 nAb and FGF21 nAb treatments) was comparable (Fig. 5a). GCGR mAb significantly lowered fasting blood glucose levels and improved glucose tolerance in *db/db* mice but the glucose-lowering effect was not abolished by adding FGF21 nAb (Fig. 5b,c), suggesting that FGF21 does not participate in the GCGR mAb-induced hypoglycaemic effect. GCGR mAb upregulated plasma glucagon and insulin levels (glucagon 5921.0±1254.0 vs 294.4±110.6 ng/l, *p*<0.001; insulin 2667.0±328.7 vs 1108.0±228.8 pmol/l, *p*<0.001), and these effects were attenuated by addition of FGF21 nAb (glucagon 1304.0±276.2 vs 5921.0±1254.0 ng/l, *p*<0.001; insulin 1463.0±201.0 vs 2667.0±328.7 pmol/l, *p*<0.01) (Fig. 5d,e), indicating that FGF21 participated in the upregulating effect of glucagon and insulin secretion induced by GCGR mAb. GCGR mAb increased islet number (1.6±0.1 vs 0.9±0.2 per mm^2^, *p*<0.01), islet area (2.4±0.2 vs 1.1±0.2%, *p*<0.001) and alpha cell number (24.0 [17.0–47.5] vs 5.0 [2.0–13.0], *p*<0.001), and these effects were attenuated by combined treatment with FGF21 nAb (islet number 1.0±0.1 vs 1.6±0.1 per mm^2^, *p*<0.05; islet area 1.6±0.2 vs 2.4±0.2%, *p*<0.05; alpha cell number 10.0 [4.0–29.0] vs 24.0 [17.0–47.5], *p*<0.001) (Fig. 5f–j). Notably, GCGR mAb increased beta cell number (48.0 [19.0–91.5] vs 9.0 [3.0–28.5], *p*<0.001), and this effect was abolished by addition of FGF21 nAb (12.0 [5.0–43.0] vs 48.0 [19.0–91.5], *p*<0.001) (Fig. 5k). These results suggest that FGF21 participates in GCGR mAb-induced beta cell regeneration.

### Liver-derived FGF21 participates in GCGR mAb-induced beta cell regeneration in vivo

**Fig. 6:**
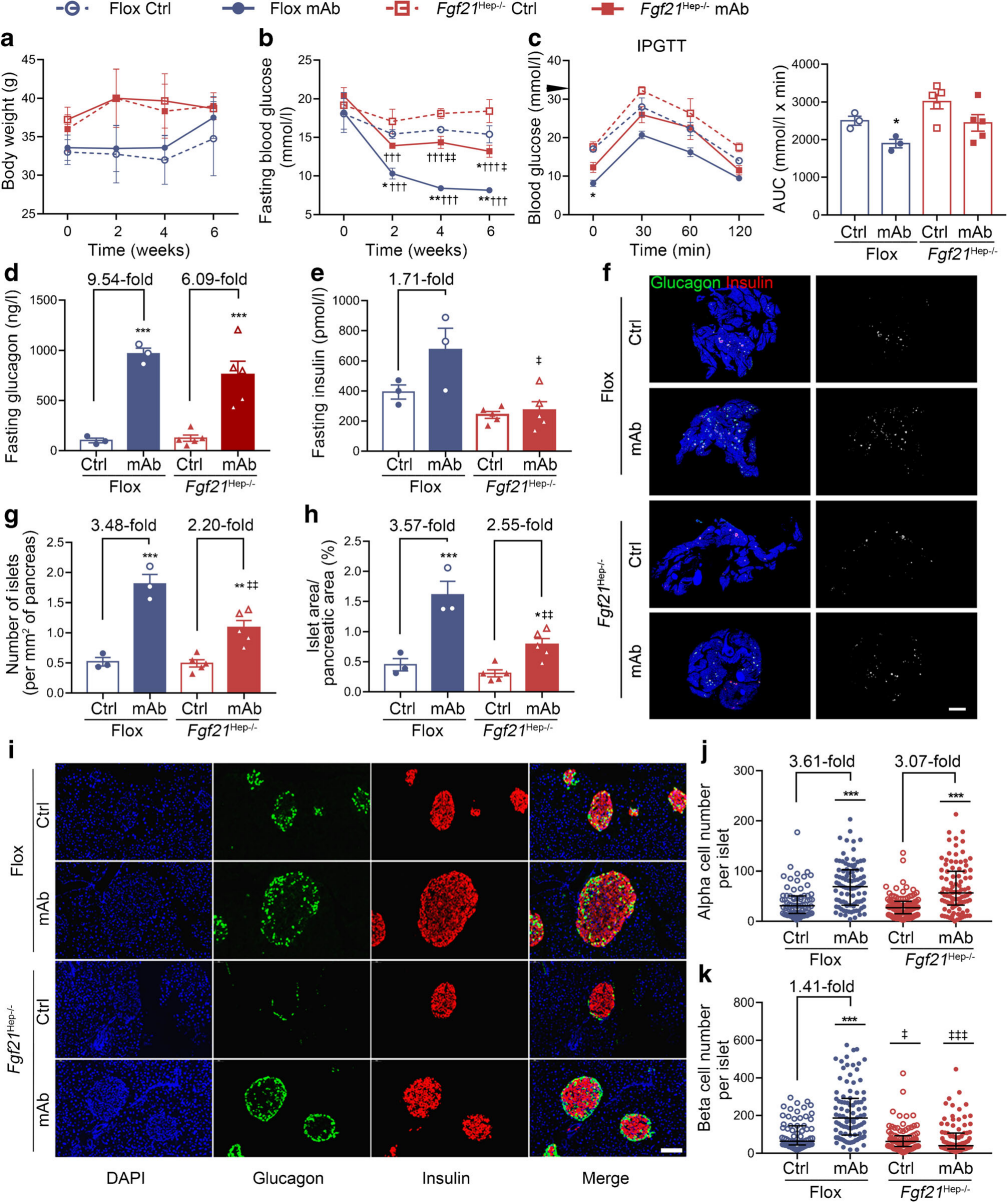
Metabolic variables, hormone levels and pancreatic histological analysis in hepatocyte-specific *Fgf21-*knockout diabetic mice treated with GCGR mAb or human IgG control for 6 weeks. (**a**) Body weight. (**b**) Fasting blood glucose. (**c**) Blood glucose during the IPGTT (black triangle indicates 33.3 mmol/l, the upper detection limit of the glucometer), and the AUC for blood glucose during the IPGTT. (**d**) Fasting plasma glucagon. (**e**) Fasting plasma insulin. (**f**) Histological analysis of the pancreas, showing representative images of the whole pancreas immunostained for glucagon and insulin together with monochrome images of immunostaining in the same tissues (cells immunolabelled positively for either glucagon or insulin are displayed in white). Scale bar, 2000 μm. (**g**, **h**) Quantification of islet number (**g**) and islet area (**h**). *n*=3 mice per group in Flox mice, and *n*=5 mice per group in hepatocyte-specific *Fgf21-*knockout (*Fgf21*^Hep−/−^) mice. (**i**) Representative images of islets immunostained for glucagon and insulin in the pancreatic tissues. (**j**, **k**) Quantification of alpha cell number (**j**) and beta cell number (**k**) per islet slice. Scale bar, 50 μm. Fifteen sections of *n*=3 mice per group in Flox mice, and 25 sections of *n*=5 mice per group in *Fgf21*^Hep−/−^ mice. Data are expressed as the mean±SEM or median (IQR). *p<0.05, **p<0.01, ***p<0.001 vs IgG control in the same genotype; ^†††^*p*<0.001 vs pre-treatment in the same group; ^‡^*p*<0.05, ^‡‡^*p*<0.01, ^‡‡‡^*p*<0.001 vs Flox group on the same treatment (ANOVA followed by post hoc Tukey–Kramer test, or Mann–Whitney test, as appropriate). Ctrl, IgG control group

Liver is the main source for the circulating FGF21 pool. To clarify whether liver-derived FGF21 had a dominant effect on beta cell regeneration, *Fgf21*^Hep−/−^ mice and their littermate Flox diabetic mice were treated with GCGR mAb or IgG. Similar to the findings in *Fgf21*^−/−^ diabetic mice, body weight was comparable among the four groups (Fig. 6a). GCGR mAb significantly lowered fasting blood glucose levels and improved glucose tolerance in Flox mice after 6 weeks of treatment but these effects were attenuated in Fgf*21*^Hep−/−^ mice (Fig. 6b,c). Fasting glucagon levels were increased similarly by GCGR mAb in Flox mice (966.5±56.5 vs 101.3±23.2 ng/l, *p*<0.001) and *Fgf21*^Hep−/−^ mice (757.0±135.6 vs 124.4±31.5 ng/l, *p*<0.001) (Fig. 6d). GCGR mAb appeared to upregulate plasma insulin level in Flox mice (673.9±142.4 vs 393.3±47.1 pmol/l, *p*=0.09), while there was no sign of the increment in *Fgf21*^Hep−/−^ mice (271.5±57.2 vs 241.0±22.9 pmol/l, *p*=0.98) (Fig. 6e). Compared with IgG control treatment, GCGR mAb treatment increased islet number (1.8±0.2 vs 0.5±0.1 per mm^2^, *p*<0.001), islet area (1.6±0.2 vs 0.5±0.1%, *p*<0.001), alpha cell number (69.0 [32.0–102.0] vs 31.0 [16.0–50.0], *p*<0.001) and beta cell number (187.0 [96.5–289.0] vs 65.0 [44.0–142.0], *p*<0.001) in Flox mice (Fig. 6f–k). In *Fgf21*^Hep−/−^ mice, although GCGR mAb increased islet number (1.1±0.1 vs 0.5±0.1 per mm^2^, *p*<0.01), islet area (0.8±0.1 vs 0.3±0.1%, *p*<0.05) and alpha cell number (57.0 [33.5–99.0] vs 27.0 [15.0–39.3], *p*<0.001), the GCGR mAb-induced fold change in islet number and islet area was much smaller than that in Flox mice. Notably, GCGR mAb did not increase beta cell number in *Fgf21*^Hep−/−^ mice (40.0 [23.5–98.5] vs 63.0 [36.5–90.5], *p*=0.08) (Fig. 6k). These results suggest that liver-derived FGF21 participates in the promoting effects of beta cell regeneration induced by GCGR mAb.

### Liver-derived FGF21 mediates the promoting effect of GCGR mAb on beta cell proliferation in diabetic mice

**Fig. 7:**
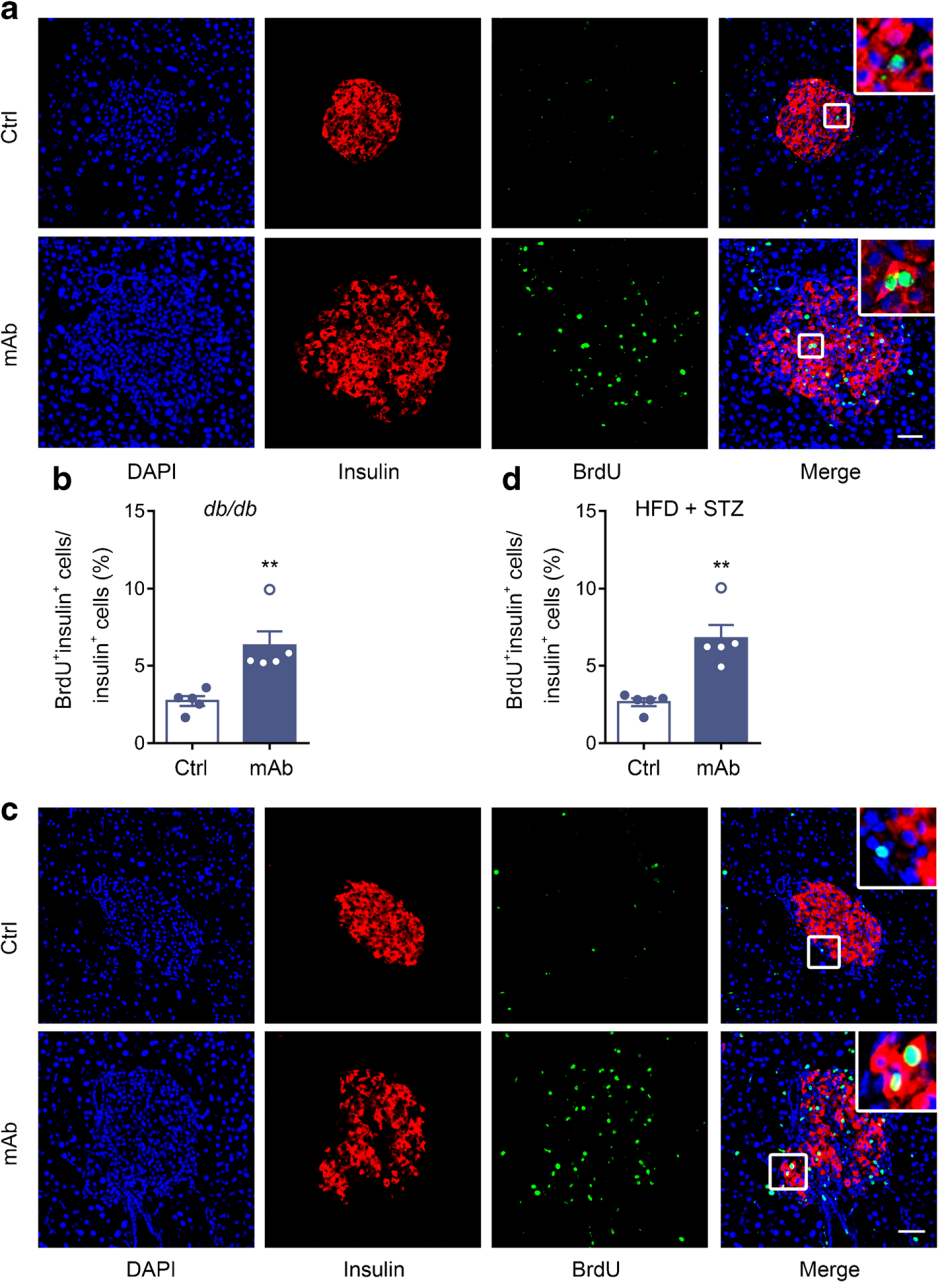
Histological analysis of beta cell proliferation in the pancreatic tissues of two mouse models of type 2 diabetes, after treatment with GCGR mAb or human IgG control for 6 weeks. (**a**, **c**) Representative photographs showing immunostaining of insulin and BrdU in *db/db* mice (**a**) and HFD+STZ-induced diabetic mice (**c**). Scale bar, 50 μm. (**b**, **d**) Quantification of BrdU^+^insulin^+^ cells (proliferating beta cells) in *db/db* mice (**b**) and HFD+STZ-induced diabetic mice (**d**). Fifteen sections of *n*=5 mice per group. Enlarged images of small boxes are shown in the corner of images. Data are expressed as the mean±SEM. **p<0.01 vs control (Student’s *t* test). Ctrl, control group

To further explore the origin of the increased number of beta cells, we examined beta cell proliferation. Histological analysis showed that the number of cells positive for both proliferating cell nuclear antigen and insulin appeared to be increased by GCGR mAb in *db/db* mice and HFD+STZ-induced diabetic mice (ESM Fig. 6). Likewise, the proportion of BrdU^+^insulin^+^ cells was also remarkably increased by GCGR mAb (*db/db* 6.3±0.9 vs 2.7±0.3%, *p*<0.01; HFD+STZ 6.8±0.9 vs 2.6±0.3%, *p*<0.01) (Fig. 7).

**Fig. 8:**
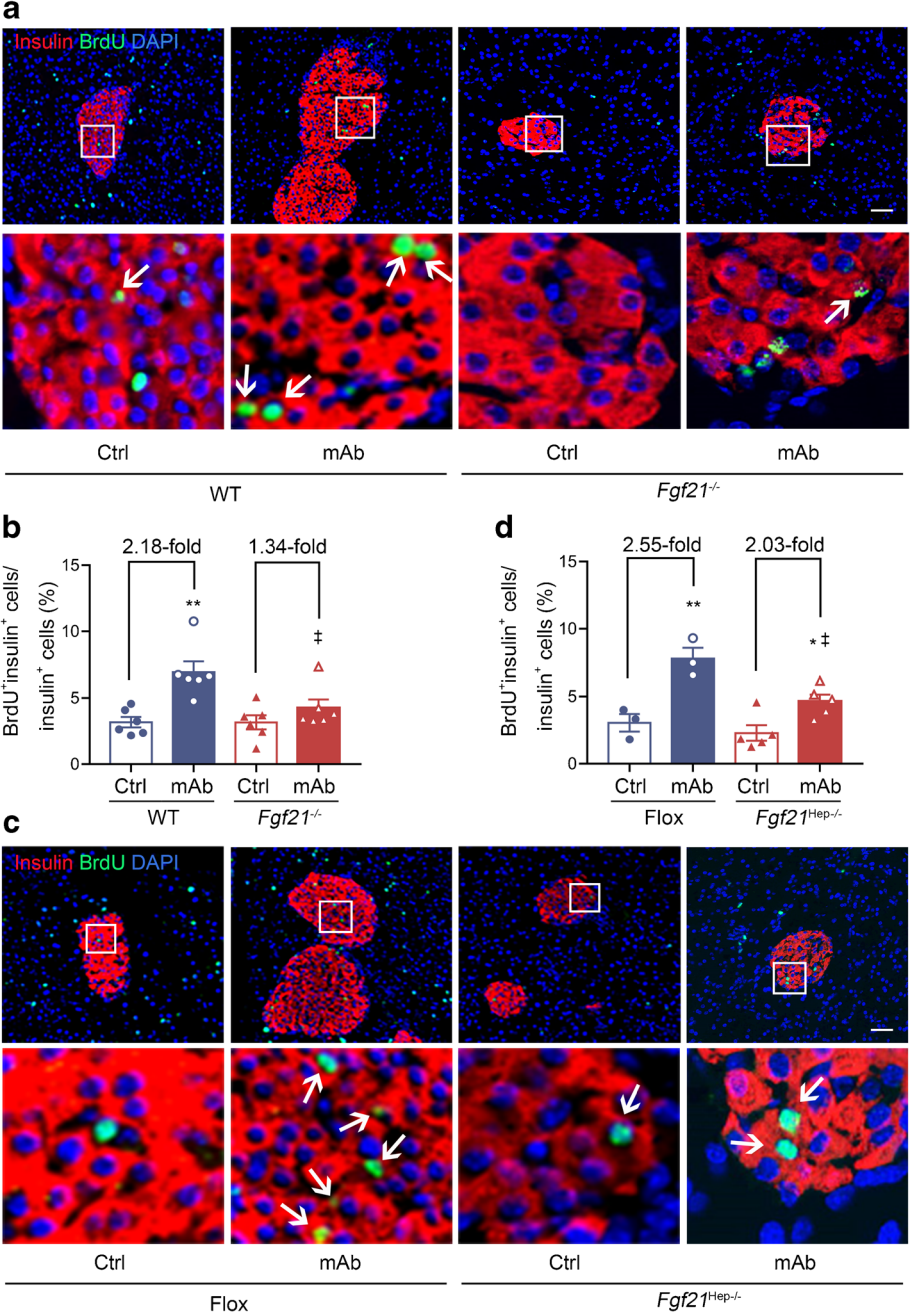
Histological analysis of beta cell proliferation in the pancreatic tissues of systemic and hepatocyte-specific *Fgf21*-knockout diabetic mice treated with GCGR mAb or human IgG control for 6 weeks. (**a**, **c**) Representative images of islets immunostained for insulin and BrdU in systemic *Fgf21*-knockout (*Fgf21*^−/−^) mice (**a**) and hepatocyte-specific *Fgf21*-knockout (*Fgf21*^Hep−/−^) mice (**c**). Scale bar, 50 μm. (**b**, **d**) Quantification of BrdU^+^insulin^+^ cells (proliferating beta cells) in the pancreas of *Fgf21*^−/−^ mice (**b**) and *Fgf21*^Hep−/−^ mice (**d**). Eighteen sections of *n*=6 mice per group in WT and *Fgf21*^−/−^ mice, nine sections of *n*=3 mice per group in Flox mice, and 15 sections of *n*=5 mice per group in *Fgf21*^Hep−/−^ mice. Enlarged images of small boxes are shown below each image. The arrows indicate BrdU^+^insulin^+^ cells. Data are expressed as mean±SEM. *p<0.05, **p<0.01 vs IgG control in the same genotype; ^‡^*p*<0.05 vs WT or Flox group on the same treatment (ANOVA followed by post hoc Tukey–Kramer test). Ctrl, control group

To investigate whether FGF21 mediated the proliferation-promoting effect, we detected BrdU^+^insulin^+^ cells in islets of *Fgf21*^−/−^ and *Fgf21*^Hep−/−^ diabetic mice treated with GCGR mAb or IgG. Histological analysis showed that the proportion of BrdU^+^insulin^+^ cells was significantly increased by GCGR mAb in WT mice (7.0±0.8 vs 3.2±0.4%, *p*<0.01) but not in *Fgf21*^−/−^ mice (4.3±0.6 vs 3.2±0.5%, *p*=0.61) (Fig. 8a,b). In both Flox and *Fgf21*^Hep−/−^ mice, GCGR mAb led to an increase in the proportion of BrdU^+^insulin^+^ cells (Flox 7.8±0.8 vs 3.1±0.7%, *p*<0.01; *Fgf21*^Hep−/−^ 4.7±0.5 vs 2.3±0.6%, *p*<0.05) but the increment in *Fgf21*^Hep−/−^ mice was not as large as that in Flox mice (Fig. 8c,d). These results indicated that liver-derived FGF21 mediates the promoting effect of GCGR mAb on beta cell self-replication in diabetic mice.

## Discussion

In this study, we demonstrated that inhibition of glucagon activity by GCGR mAb not only improved blood glucose control but also increased pancreatic beta cell mass and proliferation in mouse models of type 2 diabetes. Exposure to plasma or hepatocytes from GCGR mAb-treated mice could regulate beta cell identity in vitro. By using plasma cytokine array and liver RNA sequencing data, we observed that FGF21 levels in plasma and liver were upregulated by GCGR antagonism, and confirmed this by ELISA, quantitative RT-PCR and western blot in diabetic mice treated with GCGR mAb. Notably, addition of an FGF21 nAb diminished the GCGR mAb-mediated regulation of beta cell identity in vitro, and the effect of increased beta cell mass induced by GCGR mAb was attenuated in FGF21 nAb-treated *db/db* mice, *Fgf21*^−/−^ diabetic mice and *Fgf21*^Hep−/−^ diabetic mice. These results suggested that FGF21 contributed to GCGR mAb-induced beta cell regeneration in diabetic mice.

We and other groups have shown that GCGR mAb promotes beta cell regeneration in diabetic mice [8, 10, 12]. Therefore, the possible mechanism for beta cell regeneration induced by GCGR mAb is an important question. GCGR displays the highest abundance in liver. The metabolic phenotypes of liver-specific and systemic *Gcgr*-knockout mice are similar [15]. Our results showed that exposure to plasma or hepatocytes from the GCGR mAb-treated mice regulated beta cell identity in vitro. This suggests the existence of some soluble factors derived from the liver following the GCGR antagonism that could act on the endocrine pancreas and regulate islet regeneration. Here, we identified FGF21 as a potential mediator and proved that FGF21 levels in plasma and liver were upregulated by GCGR mAb. Similarly, previous studies reported that circulating and liver FGF21 levels are upregulated, contributing to diabetes resistance, in systemic and liver-specific *Gcgr*-knockout mice [20, 25]. In this study, we found that FGF21 was involved in glucose homeostasis, consistent with previous reports [26–28]. However, our study indicated that FGF21 had little involvement in the hypoglycaemic effect of GCGR mAb. A previous publication reported that hyperglucagonaemia and alpha cell hyperplasia remained to be observed in FGF21-deficient mice treated with REGN1193, another GCGR mAb [29]. The observations, in accordance with our findings, suggested that FGF21 had little effect on GCGR mAb-induced alpha cell hyperplasia.

Subsequently, we evaluated whether FGF21 contributed to beta cell identity in vitro and beta cell regeneration in vivo. We showed that exposure to plasma or hepatocytes from GCGR mAb-treated mice upregulated the expression of *Ins1*, *Ins2*, *Pcsk1* and *Pdx1* in a mouse beta cell line and primary mouse islets, and that these effects were attenuated by addition of an FGF21 nAb. These results suggested that FGF21 was involved in regulation of beta cell identity by GCGR mAb. Moreover, we observed that GCGR mAb increased beta cell mass and proliferation (a main source of regenerated beta cells in adult rodents [30]) in WT and Flox diabetic mice, while these effects were attenuated in *Fgf21*^−/−^ and *Fgf21*^Hep−/−^ diabetic mice, suggesting that FGF21 contributed to the promoting effect of GCGR mAb on beta cell regeneration. Consistently, a previous study reported that FGF21 promoted transdifferentiation of alpha cells to beta cells, as indicated by upregulation of PDX1 and neurogenin 3 protein levels and increment of glucagon^+^PDX1^+^ cells, in cultured beta cell-ablated islets [31]. Besides, FGF21 increased insulin content in primary islets in a rat model of type 2 diabetes [32] and increased beta cell number in a mouse model of type 2 diabetes [33, 34]. In addition, our single-cell RNA sequencing data for islet cell differentiation indicated that *FGFR1* was highly expressed in pancreatic endocrine cells, including alpha cells, beta cells and pancreatic endocrine progenitors, further supporting the concept that FGF21 participates in beta cell regeneration. Collectively, these observations suggest that FGF21 exerts beneficial effects on beta cells, and our findings highlight the importance of liver-derived FGF21 for beta cell regeneration.

A limitation of our study is that we identified only FGF21 as an important cytokine involved in GCGR mAb-induced beta cell regeneration but there might be other cytokines and metabolites that contribute to beta cell regeneration under this condition. For instance, the cytokine arrays clearly showed that other soluble factors were regulated by GCGR mAb, suggesting that they might also play a role in the process. However, FGF21 was the only common factor observed both in our cytokine array data and in the gene expression profiles. Besides, increment in glucagon-like peptide-1 (GLP-1) is one of the well-characterised consequences of GCGR antagonism [35–37]. Our previous studies found that plasma GLP-1 level, and intestinal and pancreatic GLP-1 production, were robustly upregulated by GCGR mAb [9, 38]. GLP-1 could exert various protective effects on beta cells, including promotion of beta cell proliferation and survival [39, 40]. Therefore, GLP-1 might be involved in the promoting effects of beta cell regeneration and insulin secretion induced by GCGR mAb. Investigations in systemic and pancreas-specific *Glp1r*-knockout mice will help answer this question. Furthermore, glucagon is a critical regulator of amino acid homeostasis, and amino acids in turn regulate alpha cell function and proliferation [13, 17, 41, 42]. Therefore, amino acid metabolism linked to the liver–alpha cell axis may also play an important role in GCGR mAb-induced beta cell regeneration. Another limitation is that we only used constitutive knockout mice, whereas inducible knockout mice might be more suitable to exclude the effect on islet development and avoid compensation. In addition, supplementation experiments with FGF21 in *Fgf21*^−/−^ or *Fgf21*^Hep/−^ mice are necessary to prove the involvement of FGF21 in GCGR mAb-induced beta cell regeneration. Nevertheless, we used an FGF21 nAb to realise non-genetic blockage of FGF21 and this confirmed our findings in *Fgf21*^−/−^ and *Fgf21*^Hep/−^ mice.

In conclusion, our study demonstrates that GCGR mAb not only ameliorates hyperglycaemia but also increases functional beta cell mass in mouse models of type 2 diabetes, and that these effects are at least partly mediated via liver-derived FGF21. Our study reveals a novel mechanism of beta cell regeneration regulated by alpha cell glucagon–liver FGF21 axis in diabetic mice.

## Supplementary Information

ESM (PDF 939 kb)

## Data Availability

The datasets generated and analysed during the current study are available from the corresponding authors upon reasonable request.

## Abbreviations

FGF21: Fibroblast growth factor 21
FGFR: FGF receptor
GCGR: Glucagon receptor
GLP-1: Glucagon-like peptide-1
HFD: High-fat diet
KLB: β-Klotho
mAb: Monoclonal antibody
nAb: Neutralising antibody
PDX1: Pancreatic and duodenal homeobox 1
STZ: Streptozotocin
WT: Wild-type
