# Supplementary material for: Pancreatic alpha cell glucagon–liver FGF21 axis regulates beta cell regeneration in a mouse model of type 2 diabetes
Source: Diabetologia. 2022 Nov 4;66(3):535–50. doi: 10.1007/s00125-022-05822-2 (PMC9892158; doi:10.1007/s00125-022-05822-2)
Supplement: Supplementary file 1 — (PDF 939 kb) [file 125_2022_5822_MOESM1_ESM.pdf]

Supplemental methods

Animal experiments

All animal experimental procedures were conducted at Peking University Health Science Center, and approved by the Institutional Animal Care and Use Committee. All the animals were maintained on a 12-h light/dark cycle with free access to food and water. Eight-week-old male *db/db* mice (GemPharmatech Co. Ltd., Nanjing, China) were used as a mouse model of type 2 diabetes. The littermate male *db/m* mice were included as normal controls. $n = 5$ mice per group.

The genetically modified mice were as follows: Systemic *Fgf21*-knockout (*Fgf21*$^{-/-}$) mice were established by Cyagen Bioscience Company (Suzhou, China) using CRISPR-Cas9 technique to knockout exons 1-3. The littermate wild-type (WT) mice were used as controls. Alb-cre mice (cre expression in liver cells; the Jackson Laboratory, Barr Harbor, ME, USA) and *Fgf21* Flox mice (the Jackson Laboratory) were kindly gifted by Prof. Lirui Wang from China Pharmaceutical University (Nanjing, China), and crossed to generate hepatocyte-specific *Fgf21*-knockout (*Fgf21*$^{\mathrm{Hep}-/-}$) mice [1]. The littermate *Fgf21* Flox mice were used as controls.

To generate high fat diet (HFD) + streptozotocin (STZ)-induced mouse model of type 2 diabetes, 5-week-old male C57BL/6N (Vital River Animal Center, Beijing, China) or genetically modified mice were fed with HFD (fat 60%, carbohydrate 20% and protein 20%; Research Diets, New Brunswick, NJ, USA) for 3–4 months, and then were given 75 mg/kg STZ (Sigma-Aldrich, St. Louis, MO, USA) intraperitoneally. Diabetic condition was defined if the fasting blood glucose level was ≥ 11.1 mmol/l for two

consecutive times. The mice without diabetic condition were omitted from the study. Mice were randomised into groups having similar distributions based on their body weight and blood glucose level.

To determine the effects of GCGR mAb on beta cell regeneration, *db/db* mice or HFD+STZ-induced mouse models of type 2 diabetes in C57BL/6N mice were treated for 6 weeks *via* weekly intraperitoneal injection of REMD 2.59 (a human antagonistic GCGR mAb, 5 mg/kg; REMD Biotherapeutics, Camarillo, CA, USA), or human IgG (5 mg/kg, as control). There were 5 mice in each group. To clarify the involvement of FGF21 in GCGR mAb-induced beta cell regeneration, HFD+STZ-induced mouse models of type 2 diabetes in *Fgf21*$^{-/-}$ mice and WT mice were treated with 5 mg/kg REMD 2.59 or human IgG for 6 weeks. There were 6 mice in each group. Besides, *db/db* mice were divided into four groups: 1) control group: treated with human IgG and rabbit IgG; 2) GCGR mAb group: injected with REMD 2.59 and rabbit IgG; 3) FGF21 neutralizing antibody (nAb) group: treated with FGF21 nAb (Antibody & Immunoassay Services, Hong Kong, China) and human IgG; 4) GCGR mAb + FGF21 nAb group: injected with REMD 2.59 and FGF21 nAb. REMD 2.59 and human IgG were intraperitoneally injected weekly at a dose of 5 mg/kg, while FGF21 nAb and rabbit IgG were intraperitoneally injected daily at a dose of 6 μg/day for 3 weeks. There were 6 mice in each group. To determine the effects of liver-derived FGF21 in GCGR mAb-induced beta cell regeneration, HFD+STZ-induced mouse models of type 2 diabetes in *Fgf21*$^{\text{Hep}-/-}$ mice and Flox littermates were treated with 5 mg/kg REMD 2.59 or human IgG for 6 weeks. There were 3 mice in each Flox group, and 5 mice in each *Fgf21*$^{\text{Hep}-/-}$ group. For

proliferation detection, mice were treated with 1 mg/ml 5-bromo-2'-deoxyuridine (BrdU, Sigma-Aldrich) *via* drinking water for 7 days before being sacrificed.

The high affinity (a higher affinity for GCGR than the endogenous ligand glucagon) and strong specificity (highly specific antagonistic activity against GCGR, without inhibitory effect on glucagon-like peptide-1 receptor and other receptors with similar structures) of GCGR mAb has been proved previously [2-4]. The neutralizing ability of FGF21 nAb has been reported previously [5].

Immunofluorescent staining and quantification

Pancreases were fixed with 10% (*vol/vol*) neutral-buffered formalin and embedded in paraffin, and 5-µm-thick sections were prepared. For immunofluorescence, the sections were incubated with primary antibodies at 4°C overnight and secondary antibodies for 1 h at room temperature, followed by staining with DAPI. All primary and secondary antibodies were diluted in antibody dilution buffer (Tris-HCl buffer, BSA, sodium azide; Zhongshan Biotechnology, Beijing, China). Images were captured under Leica TCS SP8 confocal fluorescence microscope (Leica Microsystems, Wetzlar, Germany) or an automatic digital slide scanner (Pannoramic MIDI, 3D HISTECH, Budapest, Hungary).

The primary antibodies were as follows: rabbit polyclonal anti-glucagon (1:800; Cell Signaling Technology, Boston, MA, USA; RRID: AB_659831), mouse monoclonal anti-insulin (1:800; Sigma-Aldrich; RRID: AB_260137), rabbit monoclonal anti-insulin (1:600; Abcam, Cambridge, UK; RRID: AB_2716761), rabbit polyclonal anti-BrdU (1:200; Abcam; RRID: AB_2813902), mouse monoclonal anti-proliferating cell nuclear antigen (1:1000; Cell Signaling Technology; RRID: AB_2160343). The secondary

antibodies were as follows: Alexa Fluor 488-conjugated AffiniPure goat polyclonal anti-rabbit IgG (H+L) (RRID: AB_2338046) and Alexa Fluor 594-conjugated AffiniPure goat polyclonal anti-mouse IgG (H+L) (RRID: AB_2338871) (both at 1:800; Jackson ImmunoResearch Laboratories, Philadelphia, PA, USA). Before testing, positive control and negative control were used to verify the antibodies.

For cell quantification in the immunofluorescent staining, 3 to 5 equally spaced sections (which covered the entire pancreas) per pancreas with 3 to 6 mice per group were imaged, and the spacing between the two adjacent sections was 200 μm. The islet number per section, and the alpha cell number and beta cell number per islet were counted manually with the positive staining of glucagon and insulin. The islet area (the glucagon-positive and insulin-positive region) was analysed by Fiji software (National Institutes of Health, Bethesda, MD, USA) [6]. For panoramic scanning, fluorescence was imaged using an automatic digital slide scanner (Pannoramic MIDI).

Primary mouse islet isolation and culture

Primary islets were isolated from chow-diet C57BL/6N mice aged 8-10 weeks as previously reported [7-9]. Briefly, the pancreas was perfused by collagenase V (Sigma-Aldrich), and individual islets were handpicked to near 100% purity under a dissecting microscope. The islets were cultured for 24 h before treatment in the RPMI 1640 medium (Invitrogen, Carlsbad, CA, USA) supplemented with 10% (*vol/vol*) foetal bovine serum (FBS; HyClone, Logan, UT, USA), 2 mmol/l GlutMax (Thermo Fisher Scientific, Waltham, MA, USA), 1 mmol/l sodium pyruvate (Thermo Fisher Scientific), and 1% (*vol/vol*) penicillin-streptomycin (Thermo Fisher Scientific).

Mouse beta cell line culture

The mouse pancreatic beta cell line Min6 cells, kindly gifted by Prof. Yiming Mu from the General Hospital of the People's Liberation Army (Beijing, China), were cultured in Dulbecco's modified eagle medium (DMEM, 25 mmol/l glucose; Invitrogen) supplemented with 15% (*vol/vol*) FBS, 2 mmol/l GlutMax and 55 μmol/l β-mercaptoethanol (Thermo Fisher Scientific). Min6 cells were verified to be of mouse origin and negative for inter-species contamination from rat or human. Mycoplasma was tested as negative using Mycoplasma PCR detection kit (Beyotime Biotechnology, Shanghai, China).

Primary mouse hepatocyte isolation and culture

Eight-week-old male C57BL/6N mice on chow-diet were treated for 6 weeks *via* intraperitoneal administration of GCGR mAb (5 mg/kg) or human IgG (5 mg/kg, as control) once a week. There were 3 mice in each group. Primary hepatocytes were isolated by nonrecirculating collagenase perfusion through the portal vein as previously reported [5] The hepatocytes were plated on 6-well plates which were coated with rat collagen type I (Sigma-Aldrich), and were then cultured in RPMI 1640 medium containing 10% (*vol/vol*) FBS for 6 h before further co-culture. In the co-culture system, the primary hepatocytes from each mouse were used independently in the experiments.

Co-culture of beta cell line or primary islets with primary hepatocytes

The co-culture system was established by the transwell chamber with 3 μm-pore size (Corning, New York, NY, USA). The primary mouse hepatocytes were plated on the lower chamber. The Min6 cells or primary mouse islets were plated on the upper

chamber. The co-culture systems were incubated for 24 h with or without FGF21 nAb (10 μg/ml; Antibody & Immunoassay Services), and the beta cell line or primary islets were collected for further analysis.

Conditional culture of beta cell line or primary islets with mouse plasma

Eight-week-old male C57BL/6N mice on chow-diet were treated for 6 weeks *via* intraperitoneal injection of GCGR mAb (5 mg/kg) or human IgG (5 mg/kg, as control) once per week. There were 6 mice in each group. Plasma was collected after a 12-h fast before sacrifice, and was stored at −80°C. In the conditional culture system, plasma from two mice in the same treatment group was pooled together. There were 3 batches of plasma in each group for the plasma-conditional culture experiments.

Min6 cells or primary mouse islets were cultured with 10% (*vol/vol*) mouse plasma and 90% culture medium with or without FGF21 nAb (10 μg/ml), and the beta cell line or primary islets were collected for further analysis.

RNA extraction, reverse transcription and quantitative RT-PCR

Total RNA was extracted with Trizol reagent (Thermo Fisher Scientific) and reversely transcribed to cDNA using a RevertAid First Strand cDNA Synthesis kit (Thermo Fisher Scientific). The quantitative RT-PCR was performed using iQ SYBR Green Supermix (Toyobo Co., Ltd., Osaka, Japan) on a QuantStudio 5 Real-Time PCR System (Thermo Fisher Scientific). Relative quantification for gene expression was calculated using the $2^{-\Delta\Delta CT}$ method, which was normalized to the internal reference, *Gapdh*. The primer sequences were summarized in ESM Table 1.

Protein extraction and western blot analysis

Total proteins from liver tissues were obtained using radioimmunoprecipitation assay lysis buffer, which contained protease inhibitor and phosphatase inhibitor (all from Applygen Technologies, Beijing, China). The denatured proteins (approximately 30 μg) were separated by 12% (*wt/vol*) SDS-PAGE electrophoresis and transferred to a nitrocellulose membrane. The membranes were incubated overnight at 4°C with the primary antibodies (both at 1:1000 dilution): rabbit anti-FGF21 (Abcam; RRID: AB_2629460) and mouse anti-GAPDH (Zhongshan Biotechnology, Beijing, China; RRID: AB_2107448). The primary antibodies were diluted in TBST with 5% BSA. Before testing, positive control and negative control were used to verify the antibodies. After three washes, the blots were incubated for 1 h with RDye 800CW-conjugated goat anti-rabbit IgG or goat anti-mouse IgG (both at 1:10,000 dilutions; LICOR Biosciences, Lincoln, NE, USA). The secondary antibodies were diluted in TBST. Protein bands were visualized with an Odyssey 290 Infrared Imaging System (LICOR Biosciences). GAPDH was used as a loading control.

References

[1] Wang L, Mazagova M, Pan C, et al. (2019) YIPF6 controls sorting of FGF21 into COPII vesicles and promotes obesity. Proc Natl Acad Sci U S A 116(30):15184-15193. https://doi.org/10.1073/pnas.1904360116

[2] Yan H, Gu W, Yang J, et al. (2009) Fully human monoclonal antibodies antagonizing the glucagon receptor improve glucose homeostasis in mice and monkeys. J Pharmacol Exp Ther 329(1):102-111. https://doi.org/10.1124/jpet.108.147009

[3] Wang MY, Yan H, Shi Z, et al. (2015) Glucagon receptor antibody completely suppresses type 1 diabetes phenotype without insulin by disrupting a novel diabetogenic pathway. Proc Natl Acad Sci U S A 112(8):2503-2508. https://doi.org/10.1073/ pnas.1424934112

[4] Gu W, Yan H, Winters KA, et al. (2009) Long-term inhibition of the glucagon receptor with a monoclonal antibody in mice causes sustained improvement in glycemic control, with reversible alpha-cell hyperplasia and hyperglucagonemia. J Pharmacol Exp Ther 331(3):871-881. https://doi.org/10.1124/jpet.109.157685

[5] Liu J, Yang K, Yang J, et al. (2019) Liver-derived fibroblast growth factor 21 mediates effects of glucagon-like peptide-1 in attenuating hepatic glucose output. EBioMedicine 41:73-84. https://doi.org/10.1016/j.ebiom.2019.02.037
[6] Schindelin J, Arganda-Carreras I, Frise E, et al. (2012) Fiji: an open-source platform for biological-image analysis. Nat Methods 9(7):676-682. https://doi.org/ 10.1038/nmeth.2019
[7] Wang L, Liu Y, Yang J, et al. (2014) GLP-1 analog liraglutide enhances proinsulin processing in pancreatic beta-cells via a PKA-dependent pathway. Endocrinology 155(10):3817-3828. https://doi.org/10.1210/en.2014-1218
[8] Wei R, Cui X, Feng J, et al. (2020) Dapagliflozin promotes beta cell regeneration by inducing pancreatic endocrine cell phenotype conversion in type 2 diabetic mice. Metabolism 111:154324. https://doi.org/10.1016/j.metabol.2020.154324
[9] Zmuda EJ, Powell CA, Hai T (2011) A method for murine islet isolation and subcapsular kidney transplantation. J Vis Exp 50(50):e2096. https://doi.org/10.3791/2096

Supplemental tables

ESM Table 1. Quantitative RT-PCR primers used for mRNA expression analysis.

Gene symbol	Gene name	Species	Gene ID	Primer sequences (5'-3')	Tm (˚C)	Product length (bp)
Fgf21	fibroblast growth factor 21	Mouse	56636	F: CTGCTGGGGGTCTACCAAG<br>R: CTGCGCCTACCACTGTTCC	62	154
Gcg	proglucagon	Mouse	14526	F: TTCATCTCATCAGGGTCCTC<br>R: GCTTATAATGCTGGTGCAAG	60	95
Ins1	insulin 1	Mouse	16333	F: TAGTGACCAGCTATAATCAGAG<br>R: ACGCCAAGGTCTGAAGGTCC	62	289
Ins2	insulin 2	Mouse	16334	F: CCCTGCTGGCCCTGCTCTT<br>R: AGGTCTGAAGGTCACCTGCT	60	213
Pcsk1	proprotein convertase subtilisin/kexin type 1	Mouse	18548	F: AGTTGGAGGCATAAGAATGCTG<br>R: GCCTTCTGGGCTAGTCTGC	60	159
Pcsk2	proprotein convertase subtilisin/kexin type 2	Mouse	18549	F: GTGTGATGGTTTTTGCGTCTG<br>R: GGGAGCTTTCGGACTCCAA	59	130
Pdx1	pancreatic and duodenal homeobox 1	Mouse	18609	F: AGGAAAACAAGAGGACCCGT<br>R: CTTCATGCGACGGTTTTGGA	60	151
Gapdh	glyceraldehyde-3-phosphate dehydrogenase	Mouse	14433	F: TGCACCACCAACTGCTTAGC<br>R: GGCATGGACTGTGGTCATGAG	60	87

Abbreviations: Tm, temperature; bp, base pair.

ESM Table 2. Cytokine array screening for the change of cytokines in the plasma of streptozotocin-induced diabetic mice treated with GCGR mAb or human IgG control.

Well number	Cytokine	Abbreviation	Gene ID	Alternative nomenclature	Mean densitometry unit of control group	Mean densitometry unit of GCGR mAb group	Fold Change (GCGR mAb /control)
J7-J8	serine (or cysteine) peptidase inhibitor, clade E, member 1	SERPINE1	18787	PAI1; PAI-1; Planh1	11428.01	24049.57	2.104440756
I21-I22	resistin	RETN	57264	ADSF; Rstn; Xcp4; Fizz3	11823.00	18440.19	1.559687896
I3-I4	serum amyloid P-component	APCS	20219	Sap	12656.31	17980.65	1.420686598
J9-J10	serine (or cysteine) peptidase inhibitor, clade F, member 1	SERPINF1	20317	Pedf; Sdf3; EPC-1; Pedfl; AI195227	11887.66	16299.18	1.371100789
H3-H4	leukemia inhibitory factor	LIF	16878	LIF	6659.36	8600.33	1.291464946
J13-J14	hepatitis A virus cellular receptor 1	HAVCR1	171283	Tim1; KIM-1; TIM-1; Timd1; AI503787	6561.97	8214.45	1.251826814
C21-C22	chemokine (C-X-C motif) ligand 2	CXCL2	20310	GROb; Gro2; Mip2; Scyb; MIP-2; Scyb2; MIP-2a; Mgsa-b; CINC-2a	2766.73	3412.46	1.233391043
I13-I14	proprotein convertase subtilisin/kexin type 9	PCSK9	100102	FH3; PC9; Narc1; HCHOLA3; AI415265; AI747682	31941.32	38946.23	1.219305589
E11-E12	growth differentiation	GDF15	23886	SBF; MIC-1; NAG-1	4839.93	5853.34	1.209385260

	factor 15						
E3-E4	fibroblast growth factor 21	FGF21	56636	FGF21	5666.88	6841.32	1.207246315
I7-I8	periostin, osteoblast specific factor	POSTN	50706	PN; PLF; Osf2; OSF-2; AI747096; A630052E07Rik	22466.36	26951.05	1.199618007
E9-E10	colony stimulating factor 3 (granulocyte)	CSF3	12985	Csfg; G-CSF; MGI-IG	3621.30	4315.32	1.191649408
H1-H2	leptin	LEP	16846	ob; obese	3747.06	4457.20	1.189519250
J15-J16	tumour necrosis factor	TNF	21926	DIF; Tnfa; TNF-a; TNFSF2; Tnlg1f; Tnfsf1a; TNFalpha; TNF-alpha	4682.96	5464.39	1.166866683
J17-J18	vascular cell adhesion molecule 1	VCAM1	22329	CD106; Vcam-1	25392.08	29592.20	1.165410632
C19-C20	chemokine (C-X-C motif) ligand 1	CXCL1	14825	KC; Fsp; N51; gro; Gro1; Mgsa; Scyb1	2803.30	3261.32	1.163386009
E1-E2	fibroblast growth factor 1	FGF1	14164	Fam; Fgfa; Dffrx; FGF-1	4465.73	5132.13	1.149225323
I11-I12	prolactin family 2, subfamily c, member 2	PRL2C2	18811	Plf; Ghd2; Plf1; MRP-1; PLF-1	6635.77	7521.13	1.133422346
J11-J12	thrombopoietin	THPO	21832	Ml; Tpo; Mgdf; Mpllg	6378.26	7031.95	1.102487199
C11-C12	haemolytic complement	HC	15139	C5; He; C5a	10436.60	11460.12	1.098070253
G11-G12	interleukin 17A	IL17A	16171	Il17; Ctla8; IL-17; Ctla-8; IL-17A	1555.35	1703.99	1.095566914
D23-D24	alpha-2-HS-	AHSG	11625	AHSG	24817.50	26804.29	1.080056009

	glycoprotein						
G5-G6	interleukin 12b	IL12B	16160	p40; Il-12b; Il12p40; Il-12p40	8953.97	9636.45	1.076220939
H5-H6	lipocalin 2	LCN2	16819	NRL; 24p3; Sip24; AW212229	12943.37	13924.66	1.075814104
J5-J6	selectin, platelet	SELP	20344	Grmp; CD62P; LECAM3; PADGEM; GMP-140	38135.61	40392.06	1.059169107
F23-F24	interleukin 7	IL7	16196	IL-7; hlb368; A630026I06Rik	8178.23	8651.86	1.057913509
G9-G10	interleukin 15	IL15	16168	IL-15; AI503618	5169.18	5445.73	1.053499781
F13-F14	interleukin 2	IL2	16183	IL-2	1387.59	1458.82	1.051333607
F3-F4	insulin-like growth factor binding protein 5	IGFBP5	16011	IGFBP-5; AI256729; AW208790; IGFBP-5P	23111.23	24052.79	1.040740367
C9-C10	coagulation factor III	F3	14066	TF; Cf3; Cf-3; CD142; AA409063	6082.81	6301.52	1.035955422
E7-E8	growth arrest specific 6	GAS6	14456	Gas-6	17539.62	18169.97	1.035938635
B21-B22	CD40 antigen	CD40	21939	IGM; p50; Bp50; GP39; IMD3; TRAP; HIGM1; T-BAM; Tnfrsf5; AI326936	3777.17	3898.94	1.032238422
E5-E6	FMS-like tyrosine kinase 3 ligand	FLT3L	14256	Ly72L; Flt3lg	8513.32	8783.01	1.031678593
F11-F12	interleukin 1 receptor antagonist	IL1RN	16181	IL-1Ra; F630041P17Rik	4416.38	4486.56	1.015890843
J3-J4	selectin, endothelial cell	SELE	20339	Elam; CD62E; ELAM-1; LECAM2; E-selectin	37638.89	38109.17	1.012494524

F9-F10	interleukin 1 beta	IL1B	16176	Il-1b; IL-1beta	3308.33	3334.40	1.007880109
I9-I10	delta like non-canonical Notch ligand 1	DLK1	13386	FA1; ZOG; pG2; DlkI; Peg9; SCP1; DLK-1; Ly107; pref-1; AW742678	9114.18	9182.08	1.007449930
G3-G4	interleukin 11	IL11	16156	IL-11	6635.19	6665.17	1.004518333
D11-D12	cystatin C	CST3	13010	CysC	18545.61	18623.80	1.004216092
I1-I2	platelet derived growth factor, B polypeptide	PDGFB	18591	Sis; PDGF-B	8982.63	8996.82	1.001579716
G1-G2	interleukin 10	IL10	16153	CSIF; IL-10	5167.65	5171.06	1.000659874
G21-G22	interleukin 33	IL33	77125	IL-33; Il1f11; NF-HEV; 9230117N10Rik	7247.93	7209.70	0.994725391
D3-D4	chemokine (C-X-C motif) ligand 10	CXCL10	15945	C7; IP10; CRG-2; INP10; IP-10; Ifi10; mob-1; Scyb10; gIP-10	5733.99	5694.86	0.993175782
H7-H8	chemokine (C-X-C motif) ligand 5	CXCL5	20311	LIX; Cxcl6; GCP-2; Scyb5; Scyb6; ENA-78; AMCF-II	38472.09	38186.13	0.992567079
H13-H14	matrix metallopeptidase 3	MMP3	17392	SL-1; SLN1; Str1; EMS-2; MMP-3; SLN-1; STR-1; Stmy1	21932.82	21750.25	0.991675945
G13-G14	interleukin 22	IL22	50929	IL-22; Iltif; IL-22a; ILTIFa	2539.29	2517.06	0.991245584
G7-G8	interleukin 13	IL13	16163	IL-13	5303.85	5237.21	0.987435542
I19-I20	regenerating islet-derived 3 gamma	REG3G	19695	AI449515; REG-3-gamma; reg III-gamma	46121.56	45314.27	0.982496472
E13-E14	colony stimulating factor 2	CSF2	12981	CSF; Csfgm; GMCSF; Gm-CSf; MGI-IGM	2668.75	2618.90	0.981320843

	(granulocyte-macrophage)						
A3-A4	adiponectin, C1Q and collagen domain containing	ADIPOQ	11450	Ad; APN; Acdc; apM1; 30kDa; GBP28; adipo; Acrp30	36987.34	36115.06	0.976416796
D1-D2	chemokine (C-X-C motif) ligand 9	CXCL9	17329	CMK; Mig; MuMIG; Scyb9; crg-10; BB139920	4800.80	4687.46	0.976391435
H19-H20	secreted phosphoprotein 1	SPP1	20750	OP; 2AR; Bsp; Eta; Opn; Ric; BNSP; BSPI; Opnl; Apl-1; ETA-1; Spp-1	42296.95	41172.68	0.973419596
G15-G16	interleukin 23, alpha subunit p19	IL23A	83430	p19; IL-23	3635.95	3523.22	0.968995723
H9-H10	colony stimulating factor 1 (macrophage)	CSF1	12977	op; Csfm; MCSF; C87615	18783.68	18193.03	0.968555150
E23-E24	insulin-like growth factor binding protein 2	IGFBP2	16008	IBP-2; Igfbp-2; AI255832; mIGFBP-2	37602.54	36305.67	0.965511106
B11-B12	chemokine (C-C motif) ligand 19	CCL19	24047	ELC; CKb11; MIP3B; Scya19; exodus-3	7027.74	6748.33	0.960241842
C17-C18	chemokine (C-X3-C motif) ligand 1	CX3CL1	20312	CX3C; Cxc3; Scyd1; ABCD-3; AB030188; AI848747; D8Bwg0439e	4746.28	4554.69	0.959633650
H11-H12	matrix metallopeptidase 2	MMP2	17390	GelA; Clg4a; MMP-2	26087.12	24876.35	0.953587441
F5-F6	insulin-like growth factor binding	IGFBP6	16012	IGFBP-6	34947.89	33219.43	0.950541792

	protein 6						
B9-B10	chemokine (C-C motif) ligand 17	CCL17	20295	Tarc; Abcd-2; Scya17; Scya17l	6687.42	6329.60	0.946493566
D21-D22	collagen, type XVIII, alpha 1	COL18A1	12822	COL18A1	28000.46	26484.05	0.945843390
B7-B8	chemokine (C-C motif) ligand 12	CCL12	20293	MCP-5; Scya12	9246.34	8704.57	0.941407086
D17-D18	epidermal growth factor	EGF	13645	AI790464	12539.46	11801.07	0.941114689
I17-I18	retinol binding protein 4, plasma	RBP4	19662	Rbp-4	21441.18	20024.05	0.933906156
F19-F20	interleukin 5	IL5	16191	IL-5	5616.17	5202.50	0.926343042
C3-C4	CD160 antigen	CD160	54215	By55; AU045688	7211.99	6664.45	0.924079207
F17-F18	interleukin 4	IL4	16189	IL-4; BSF-1	13389.83	12285.20	0.917502313
F15-F16	interleukin 3	IL3	16187	BPA; PSF; HCGF; Il-3; MCGF; Csfmu	2311.54	2111.45	0.913438660
B3-B4	chemokine (C-C motif) ligand 6	CCL6	20305	c10; MRP-1; Scya6	42889.04	39065.96	0.910861143
F1-F2	insulin-like growth factor binding protein 3	IGFBP3	16009	IGFBP-3; IGgfbp3; AI649005	28297.03	25764.30	0.910494847
E15-E16	hepatocyte growth factor	HGF	15234	SF; NK1; NK2; HGF/SF; SF/HGF; C230052L06Rik	6299.68	5628.78	0.893502527
E21-E22	insulin-like growth factor binding protein 1	IGFBP1	16006	COL18A1	47978.89	42737.01	0.890746118
D13-D14	dickkopf WNT signalling pathway	DKK1	13380	mdkk-1	8109.74	7216.88	0.889902759

	inhibitor 1						
F7-F8	interleukin 1 alpha	IL1A	16175	IL-1a	8672.47	7708.17	0.888809071
C15-C16	C-reactive protein, pentraxin-related	CRP	12944	AI255847	36540.32	32357.66	0.885533022
A7-A8	angiopoietin 1	ANGPT1	11600	1110046O21Rik, Ang-1, Ang1	14813.86	13059.26	0.881556866
B5-B6	chemokine (C-C motif) ligand 11	CCL11	20292	Scya11; eotaxin	21486.93	18851.16	0.877331475
H23-H24	thymidine phosphorylase	TYMP	72962	TP; Ecgf1; PDECGF; Pdgfec; PD-ECGF; 2900072D10Rik	6551.09	5737.65	0.875831350
A5-A6	amphiregulin	AREG	11839	AR; Mcub; Sdgf	6832.27	5948.53	0.870652067
G19-G20	interferon lambda 2/interferon lambda 3	IFNL2/IFNL3	330496/338374	Il28a; IL-28A; EG330496/Il28; IFL-1; Il28b; IL-28B; INF-alpha; INF-lambda	14044.74	12073.83	0.859669172
D15-D16	dipeptidyl peptidase 4	DPP4	13482	Cd26; THAM; Dpp-4	25860.33	22213.13	0.858965450
E17-E18	intercellular adhesion molecule 1	ICAM1	15894	CD54; Ly-47; Icam-1; MALA-2	34642.84	29738.95	0.858444342
J19-J20	vascular endothelial growth factor A	VEGFA	22339	Vpf; VEGF	9283.90	7964.81	0.857916393
G17-G18	interleukin 27	IL27	246779	p28; Il30; IL-27; IL-27p28	6848.99	5873.07	0.857508917
A9-A10	angiopoietin 2	ANGPT2	11601	Ang2; Agpt2; Ang-2	38192.44	32509.84	0.851211392
C13-C14	complement factor D (adipsin)	CFD	11537	DF; Adn	36482.80	30994.52	0.849565275

C7-C8	chitinase-like 1	CHIL1	12654	Gp39; Brp39; Chi3l1; AW208766	25513.82	21649.64	0.848545612
I5-I6	pentraxin related gene	PTX3	19288	TSG-14; AI607804	31311.75	26498.12	0.846267615
C5-C6	retinoic acid receptor responder (tazarotene induced) 2	RARRES2	71660	AI303516; 0610007L05Rik	25505.20	21486.73	0.842445070
D9-D10	chemokine (C-X-C motif) ligand 16	CXCL16	66102	SR-PSOX; Zmynd15; AV290116; BB024863; CXCL16v1; CXCL16v2; b2b498Clo; 0910001K24Rik	8648.92	7263.31	0.839793870
F21-F22	interleukin 6	IL6	16193	IL6	6477.72	5368.17	0.828712880
D5-D6	chemokine (C-X-C motif) ligand 11	CXCL11	56066	Ip9; H174; Itac; b-R1; Cxc11; I-tac; Scyb11; Scyb9b; betaR1	7568.12	6253.36	0.826276539
J21-J22	WNT1 inducible signalling pathway protein 1	WISP1	22402	CCN4; Elm1; AW146261	7935.29	6437.17	0.811207908
A11-A12	angiopoietin-like 3	ANGPTL3	30924	hypl	37572.13	30097.20	0.801051205
B17-B18	chemokine (C-C motif) ligand 22	CCL22	20299	ABCD-1, DCBCK, MDC, Scya22	5084.97	4069.82	0.800362637
E19-E20	interferon gamma	IFNG	15978	Ifg; IFN-g	7040.24	5616.46	0.797765417
I15-I16	advanced glycosylation end product-specific	AGER	11596	RAGE	12722.44	10073.44	0.791785224

	receptor						
H17-H18	myeloperoxidase	MPO	17523	mKIAA4033	27304.61	21600.17	0.791081433
A21-A22	chemokine (C-C motif) ligand 5	CCL5	20304	SISd; Scya5; RANTES; TCP228; MuRantes	3611.75	2834.35	0.784758081
D19-D20	endoglin	ENG	13805	Endo; CD105; AI528660; AI662476; S-endoglin	15270.24	11977.15	0.784345891
B19-B20	CD14 antigen	CD14	12475	CD14	8314.68	6322.47	0.760398476
H15-H16	Matrix metallopeptidase 9	MMP9	17395	Clg4b; Gel B; MMP-9; B/MMP9; AW743869; pro-MMP-9	11181.92	8495.85	0.759784545
G23-G24	low density lipoprotein receptor	LDLR	16835	Hlb301	22569.80	16590.17	0.735060568
H21-H22	tumour necrosis factor receptor superfamily, member 11b (osteoprotegerin)	TNFRSF11B	18383	Opg; TR1; OCIF	8659.18	5867.37	0.677589564
A13-A14	tumour necrosis factor (ligand) superfamily, member 13b	TNFSF13B	24099	BAFF; BLyS; TALL1; THANK; zTNF4; TALL-1; Tnlg7a; TNFSF20; D8Ertd387e	35076.33	23752.45	0.677164629
D7-D8	chemokine (C-X-C motif) ligand 13	CXCL13	55985	BLC; Angie; BCA-1; BLR1L; ANGIE2; Scyb13; 4631412M08Rik	42384.47	28195.58	0.665233752

B15-B16	chemokine (C-C motif) ligand 21A (serine)	CCL21A	18829	ALP; SLC; plt; CKb9; Tca4; 6Ckine; Scya21; 6CKBAC2; SCYA21a; Scya21b; AW987545	35270.61	22752.22	0.645075886
A19-A20	chemokine (C-C motif) ligand 3/chemokine (C-C motif) ligand 4	CCL3/CCL4	20302/20303	Mip1a; Scya3; G0S19-1; AI323804; MIP1-(a); LD78alpha; MIP-1alpha; MIP1-alpha/Act-2; Mip1b; Scya4; MIP-1B; AT744.1	3664.53	2216.49	0.604849735
A15-A16	CD93 antigen	CD93	17064	Ly68; AA4.1; C1qr1; C1qrp; AA145088; AW555904; 6030404G09Rik	40555.09	24024.00	0.592379403
A17-A18	chemokine (C-C motif) ligand 2	CCL2	20296	JE; HC11; MCAF; MCP1; MCP-1; Scya2; Sigje; SMC-CF; AI323594	4111.74	2367.32	0.575746521
B13-B14	chemokine (C-C motif) ligand 20	CCL20	20297	CKb4; LARC; ST38; MIP3A; MIP-3A; Scya20; MIP-3(a); exodus-1	34588.73	8286.37	0.239568495

Supplemental figures

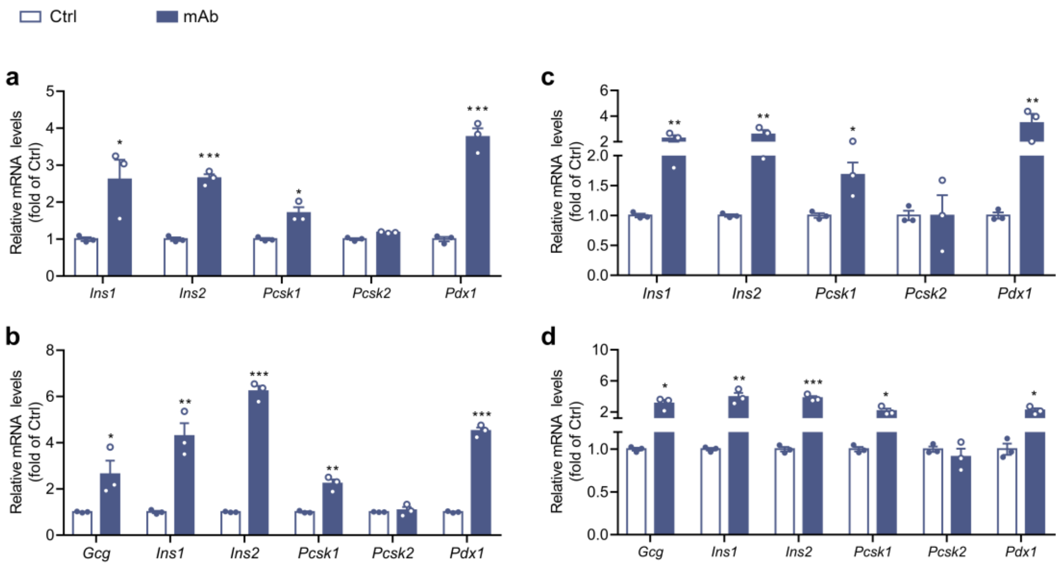

ESM Fig. 1 Regulation of beta cell identity in mouse beta cell line Min6 cells and primary mouse islets by exposure to plasma or hepatocytes from the GCGR mAb-treated non-diabetic C57BL/6N mice. (**a, b**) Relative gene expression levels in Min6 cells (**a**) and primary mouse islets (**b**) after exposed to plasma-conditional culture for 24 h. (**c, d**) Relative gene expression levels in Min6 cells (**c**) and primary mouse islets (**d**) after exposed to hepatocyte co-culture systems for 24 h. Data represent 3 independent experiments and are expressed as mean ± SEM. *$P < 0.05$, **$P < 0.01$, ***$P < 0.001$ vs control (Student's *t* test). Ctrl, control group

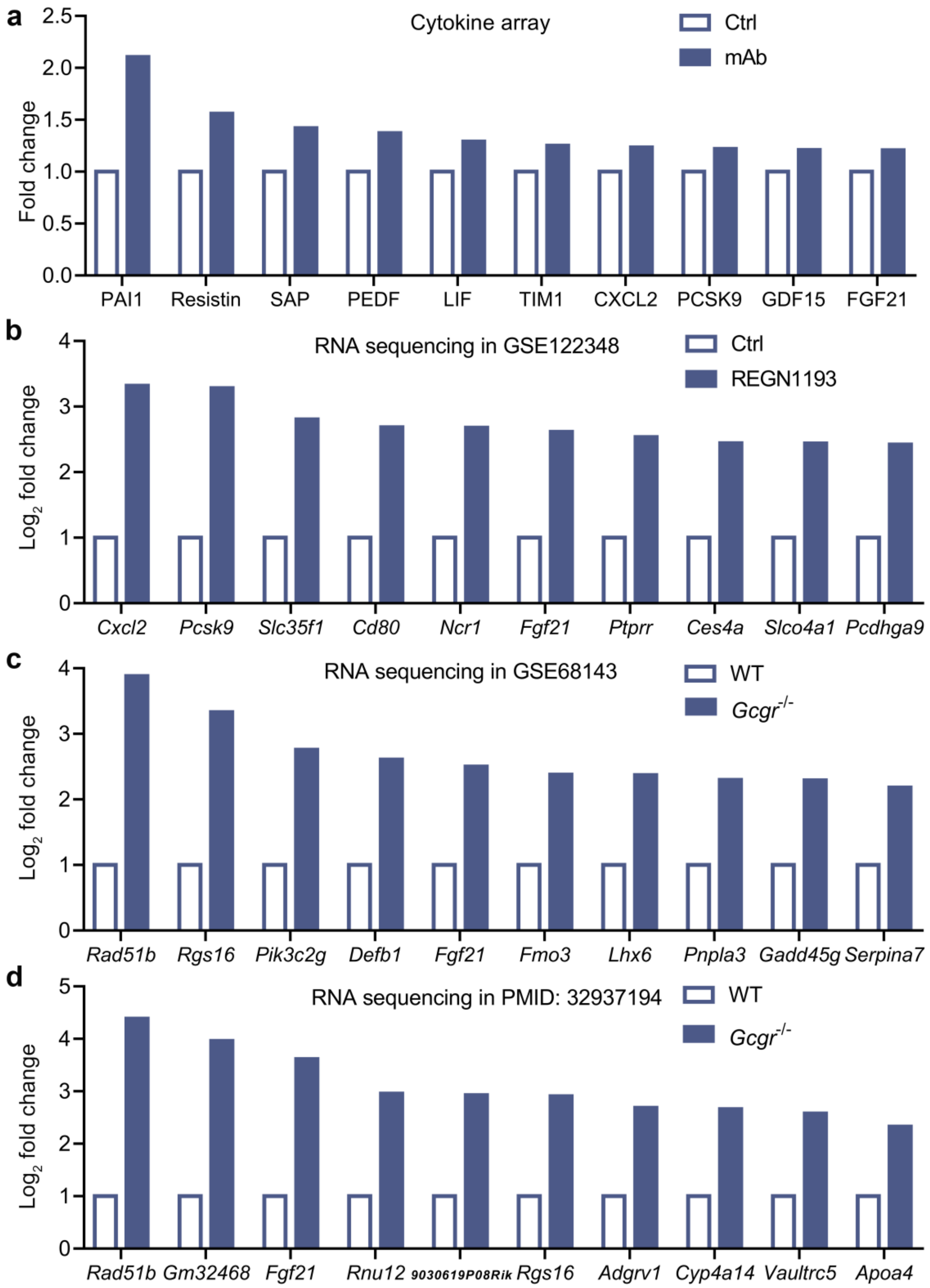

ESM Fig. 2 Candidate protein or gene screening from the plasma cytokine array or liver RNA sequencing data in the GCGR antagonistic and control mice. (**a**) The fold change of the top 10 upregulated proteins in the GCGR mAb- and human IgG control-treated STZ-

induced diabetic mice in our cytokine array screening. (**b**) The log fold change of the top 10 upregulated genes in the GCGR antagonist REGN1193-treated mice and control mice. The original data were from GSE122348. (**c**) The log fold change of the top 10 upregulated genes in the *Gcgr*-knockout (*Gcgr*$^{-/-}$) mice and wild-type (WT) mice. The original data were from GSE68143, and the data were permitted to be used by Prof. Lale Ozcan. (**d**) The log fold change of the top 10 upregulated genes in the *Gcgr*$^{-/-}$ mice and WT mice. The original data were from the published article (Winther-Sorensen M et al (2020) Mol Metab 42:101080. PMID 32937194), and the data were permitted to be used by Prof. Nicolai J. Wewer Albrechtsen. Ctrl, control group

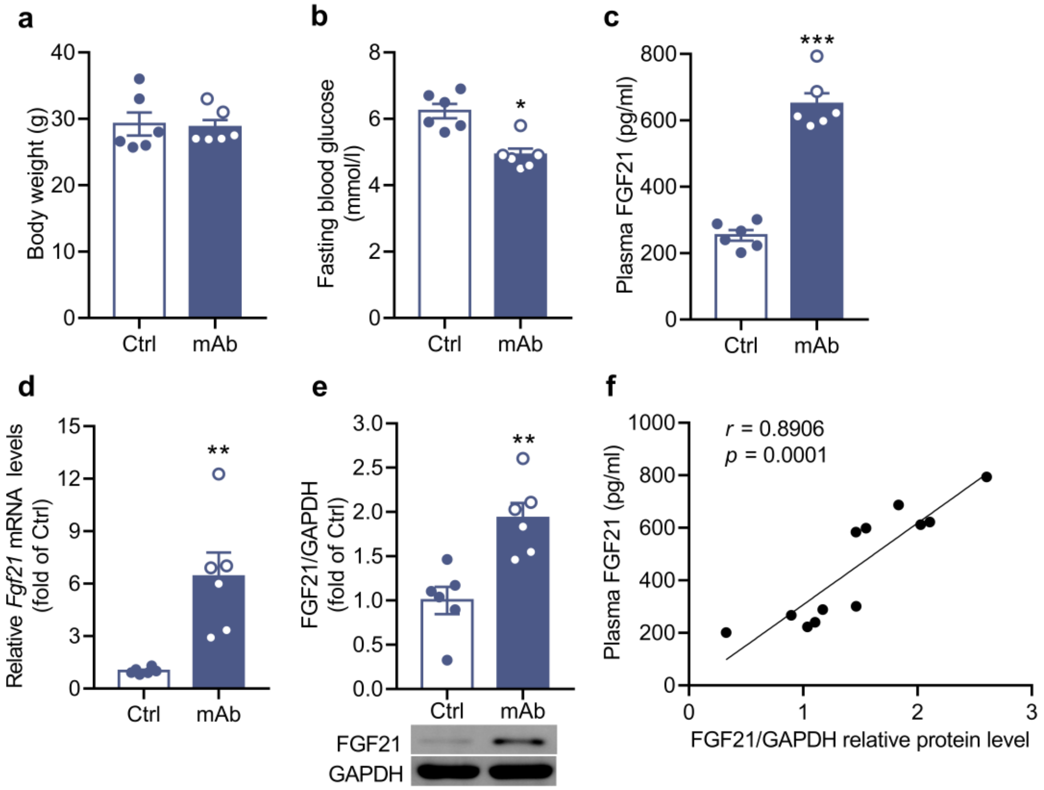

ESM Fig. 3 Metabolic variables and FGF21 levels in non-diabetic C57BL6/N mice treated with GCGR mAb or human IgG control for 6 weeks. (**a**) Body weight. (**b**) Fasting blood glucose. (**c**) Fasting plasma FGF21. (**d**) Liver *Fgf21* mRNA levels. (**e**) Liver FGF21 protein levels. (**f**) Correlation of plasma FGF21 concentrations with hepatic FGF21 protein levels. $n = 6$ mice per group. Data represent the mean ± SEM. *$P < 0.05$, **$P < 0.01$, ***$P < 0.001$ vs control (Student's t test). Ctrl, control group

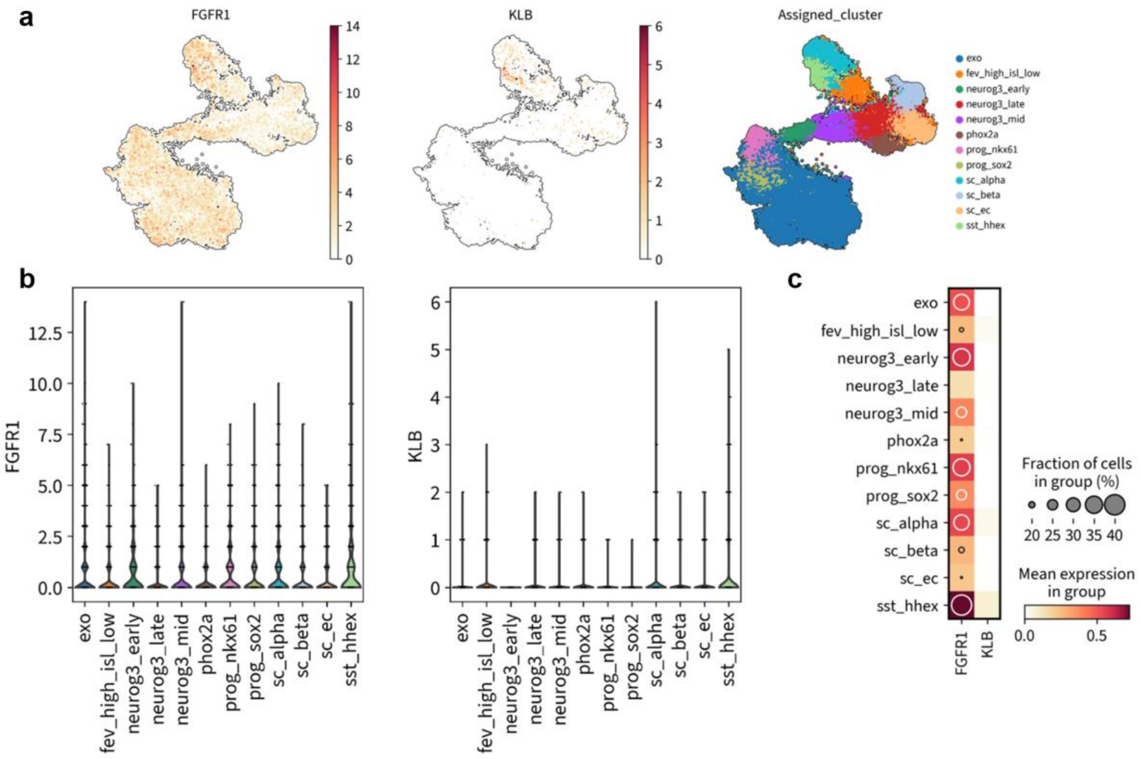

ESM Fig. 4 Single-cell RNA sequencing analysis of *FGFR1* and *β-klotho* expression in the stage-5 of *in vitro* beta cell differentiation from human embryonic stem cells. (**a**) Expression of *FGFR1* and *β-klotho* (*KLB*) in each major cell type in stage-5 time-course data. (**b**) Expression of *FGFR1* and *KLB* in each major cell type in stage-5, shown as violin plots. (**c**) Gene expression of *FGFR1* and *KLB* for each subpopulation of major cell types. Shading displays mean expression (z-normalized TPM) and diameter denotes fractional expression. The original data were from GSE114412.

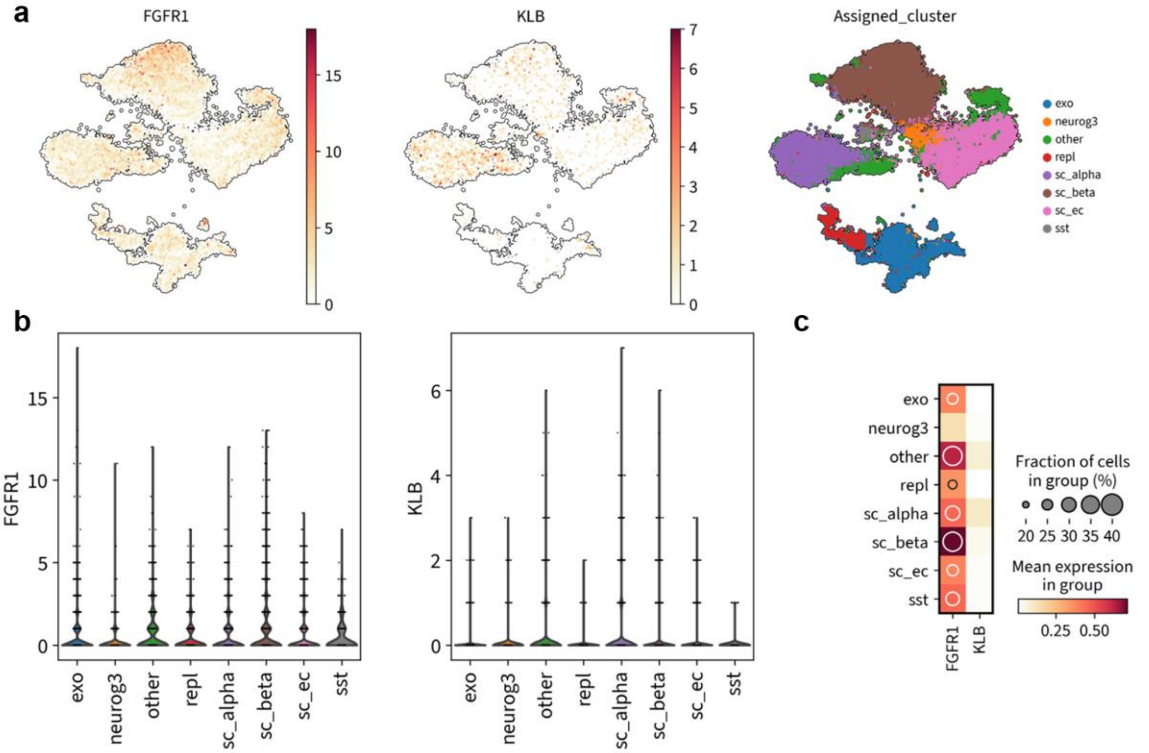

ESM Fig. 5 Single-cell RNA sequencing analysis of *FGFR1* and *β-klotho* expression in the stage-6 of *in vitro* beta cell differentiation from human embryonic stem cells. (**a**) Expression of *FGFR1* and *β-klotho* (*KLB*) in each major cell type in stage-6 time-course data. (**b**) Expression of *FGFR1* and *KLB* in each major cell type in stage-6, shown as violin plots. (**c**) Gene expression of *FGFR1* and *KLB* for each subpopulation of major cell types. Shading displays mean expression (z-normalized TPM) and diameter denotes fractional expression. The original data were from GSE114412.

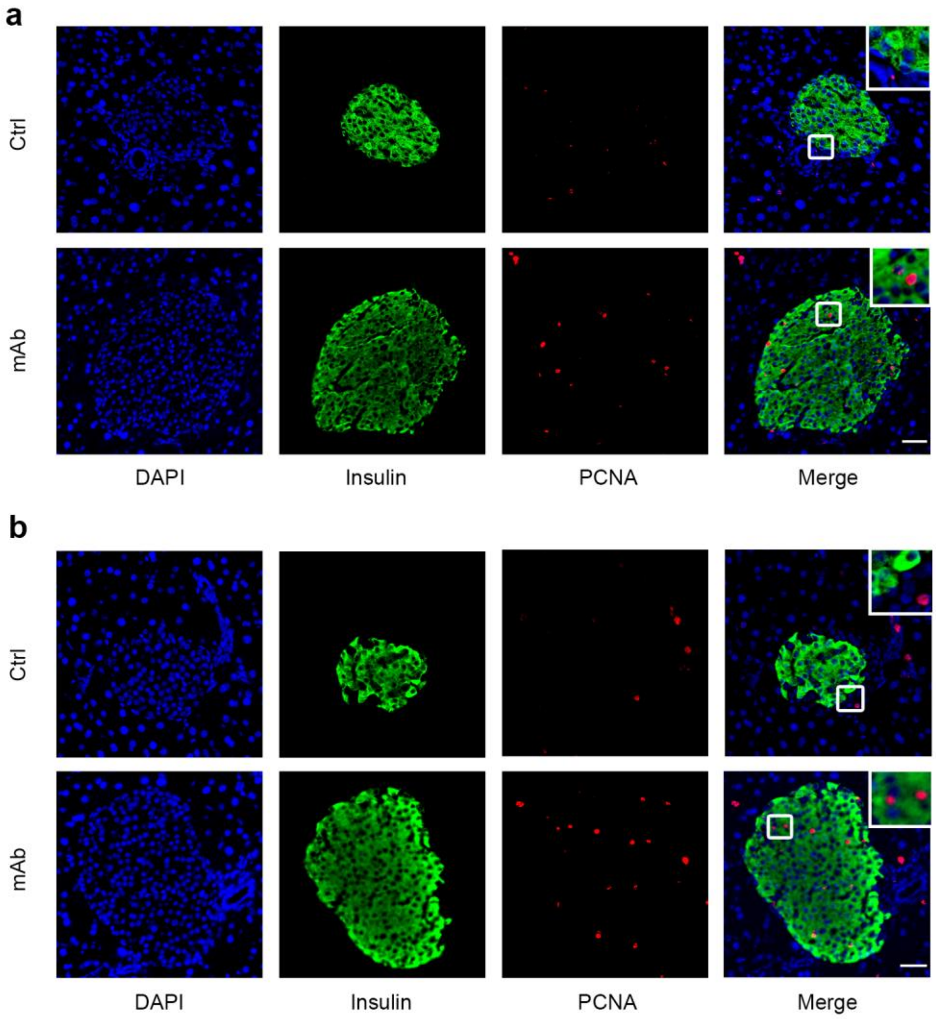

ESM Fig. 6 Histological analysis of beta cell proliferation in the pancreatic tissues of two mouse models of type 2 diabetes treated with GCGR mAb or human IgG control for 6 weeks. (**a, b**) Representative photograph showing immunostaining of insulin and proliferating cell nuclear antigen (PCNA) in *db/db* mice (**a**) and HFD+STZ-induced mouse models of type 2 diabetes (**b**). Enlarged images of small boxes are shown in the corner of images. Scale bar, 50 μm.
